# Overcoming Resistance of Cancer Cells to PARP-1 Inhibitors with Three Different Drug Combinations

**DOI:** 10.1371/journal.pone.0155711

**Published:** 2016-05-19

**Authors:** Michal Yalon, Liron Tuval-Kochen, David Castel, Itai Moshe, Inbal Mazal, Osher Cohen, Camila Avivi, Kineret Rosenblatt, Sarit Aviel-Ronen, Ginette Schiby, Joachim Yahalom, Ninette Amariglio, Raphael Pfeffer, Yaacov Lawrence, Amos Toren, Gideon Rechavi, Shoshana Paglin

**Affiliations:** 1 Department of Pediatric Hematology-Oncology, Safra Children's Hospital, Sheba Medical Center, Ramat Gan 52621, Israel; 2 Cancer Research Center, Sheba Medical Center, Ramat-Gan 52621, Israel; 3 Sackler School of Medicine, Tel Aviv University, Tel Aviv 69978, Israel; 4 Neufeld Cardiac Research Institute, Sheba Medical Center, Ramat Gan 52621, Israel; 5 Department of Surgery, Sheba Medical Center, Ramat-Gan 52621, Israel; 6 Department of Pathology, Sheba Medical Center, Ramat-Gan 52621, Israel; 7 Department of Radiation Oncology, Memorial Sloan Kettering, New York 10021, United States of America; Northwestern University Feinberg School of Medicine, UNITED STATES

## Abstract

Inhibitors of poly[ADP-ribose] polymerase 1 (PARPis) show promise for treatment of cancers which lack capacity for homologous recombination repair (HRR). However, new therapeutic strategies are required in order to overcome innate and acquired resistance to these drugs and thus expand the array of cancers that could benefit from them. We show that human cancer cell lines which respond poorly to ABT-888 (a PARPi), become sensitive to it when co-treated with vorinostat (a histone deacetylase inhibitor (HDACi)). Vorinostat also sensitized PARPis insensitive cancer cell lines to 6-thioguanine (6-TG)–a drug that targets PARPis sensitive cells. The sensitizing effect of vorinostat was associated with increased phosphorylation of eukaryotic initiation factor (eIF) 2α which in and of itself increases the sensitivity of cancer cells to ABT-888. Importantly, these drug combinations did not affect survival of normal fibroblasts and breast cells, and significantly increased the inhibition of xenograft tumor growth relative to each drug alone, without affecting the mice weight or their liver and kidney function. Our results show that combination of vorinostat and ABT-888 could potentially prove useful for treatment of cancer with innate resistance to PARPis due to active HRR machinery, while the combination of vorinostat and 6-TG could potentially overcome innate or acquired resistance to PARPis due to secondary or reversal BRCA mutations, to decreased PARP-1 level or to increased expression of multiple drug resistant proteins. Importantly, drugs which increase phosphorylation of eIF2α may mimic the sensitizing effect of vorinostat on cellular response to PARPis or to 6-TG, without activating all of its downstream effectors.

## Introduction

It has recently been discovered that breast, ovarian and prostate cancer cells which carry bi-allelic mutations for breast cancer susceptibility gene (BRCA) 1 or BRCA 2 or that are phosphatase and tensin homolog deficient (and therefore unable to express RAD51), are extremely sensitive to inhibitors of PARPis and to deletion of PARP-1 [1,2]. These findings reinforced the notion that impaired capacity for HRR results in sensitivity to PARPis.

The human PARP family consists of 17 enzymes each of which is the product of a different gene. The enzymes all possess conserved catalytic domains and DNA binding domains. PARP-1 binds to stalled replication forks as well as to single strand breaks (SSBs) that may form spontaneously, during base excision repair or in response to DNA damaging agents. Binding to DNA lesions activates PARP-1 leading to addition of poly(ADP-ribose) moieties from NAD^+^ to proteins involved in DNA repair and replication including PARP-1 itself and to histones in the vicinity of PARP-1's binding sites. Accumulation of poly(ADP-ribose) leads to recruitment of DNA repair proteins. At SSBs the main role of PARP-1 is to recruit x-ray repair cross-complementing protein 1 (XRCC1), while at stalled replication forks its activity involves the recruitment of checkpoint kinase 1 (CHK1) [3–5]. PARP-2 is also activated by DNA damage and has a role in suppressing spontaneous formation of DNA toxic lesions, however its relative abundance and catalytic activities are lower than those of PARP-1 [3]. While PARP-1 -/- or PARP-2 -/- mice are viable and healthy, the doubly-knockout mice die in utero, suggesting that some of the roles played by each of these PARPs are essential and redundant [1,3]. Pharmacological inhibition of PARP-1 activity or its deletion inhibits repair of SSB, which in the absence of functional HRR may lead to formation of unrepaired double strand breaks (DSBs) during replication, to replication fork collapse and cell death [1,3,4]. It is therefore clear why PAPRis target cells with mutated BRCA or that are otherwise impaired in their capacity for HRR. Unfortunately, in addition to their limited target population, development of resistance to PARPis is a major concern. Secondary BRCA mutations, which restore the BRCA's open reading frame (ORF) and functional C terminal as well as increased expression of multidrug resistance (MDR) 1 or decreased expression of PARP-1 all lead to loss of sensitivity to PARP-1 inhibitors [1,3]. Also, decreased expression of 53BP1 has been demonstrated to abrogate sensitivity of BRCA1 mutated cells to PARPis [6]. It is therefore important to find ways to overcome resistance to PARPis regardless of it being acquired or innate.

Relevant to this notion are the findings that HDACis decrease the level of HRR proteins and at 5 μM, vorinostat (the first HDACi to be FDA approved for cancer treatment) dramatically reduces the level of these proteins in cancer cells but not in normal cells [7,8]. Therefore, vorinostat is expected to sensitize cancer cells to treatment with PARPis, regardless of their innate capacity for DNA repair. Also, the activity of NF-kB and CHK1 which is associated with resistance to vorinostat [7] is dependent on PARP-1 activity [5,9]. Hence, vorinostat and PARPis are expected to mutually enhance the anti-neoplastic effect of each other. Indeed, while this work was in progress several laboratories reported that HDACis sensitized cultured cancer cells to PARPis [10–14].

In addition to PARPis, the anti-metabolite 6-thioguanine (6-TG) also targets cells with BRCA mutation or with other deficiencies in their capacity for HRR [15]. 6-TG is currently used in treatment of acute lymphoblastic leukemia. Within cells 6-TG is metabolized into 6-TG nucleotide which is incorporated into DNA during replication. Following its incorporation into DNA the 6-TG nucleotide undergoes S-methylation, activating mismatch repair (MMR) and triggering formation of DSBs that require HR for repair [16]. In addition, 6-TG-dependent accumulation of reactive oxygen species leads to oxidation of the incorporated 6-TG and to MMR-independent formation of inter-strand cross-links (ICL) [17] which requires the combined action of Fanconi anemia (FA) and HRR pathways for their repair [17,18]. Interestingly, cells that became resistant to PARPis due to secondary BRCA2 mutation were still sensitive to 6-TG [15]. Apparently, secondary mutations did not enable the BRCA proteins to repair MMR independent lesions following formation of ICL [15,18]. As it turned out, BRCA1 has a unique role in FA-dependent repair of ICL, which cannot be fulfilled by BRCA1 with a secondary mutation that abrogated sensitivity to PARPis [18]. The ability of vorinostat to dramatically decrease the level of DNA repair proteins including that of BRCA1 is likely to enhance the effect of 6-TG by decreasing both FA-dependent and HR repair. Also, neither vorinostat nor 6-TG are multiple drug resistant (MDR) 1 substrates [7,15] and therefore their combined action should not be affected by a possible increase in the level of MDR1. Lastly, vorinostat-induced increase in phosphorylation of eIF2α is also expected to contribute to inhibition of HRR [19] thus leading to enhanced cellular response to both PARPis and 6-TG.

The experiments presented here demonstrate that cells which respond poorly to PARP-1 inhibitor—ABT- 888 –become sensitive to it in the presence of vorinostat. Similarly, vorinostat enhanced the response of cancer cells to 6-TG. Under our experimental conditions the combined treatment of vorinostat and ABT-888 showed only a moderate effect on the level of BRCA1 and none on RAD51, but did lead to increased phosphorylation of eIF2α. Experiments with the pharmacological inhibitor of eIF2α dephosphorylation—salubrinal—as well as with the phosphomimetic S51D and the non-phosphorylatable S51A variants of eIF2α showed that increased phosphorylation of eIF2α –in and of itself—sensitizes cancer cells to ABT-888.

Finally, the combined treatments of vorinostat and ABT-888 or vorinostat and 6-TG significantly increased the inhibition of xenograft tumors growth relative to each drug alone without affecting kidney or liver functions or causing any significant weight loss.

## Materials and Methods

### Cell culture

Human breast MDA-MB-231, BT-549 and MCF-7 and human glioblastoma U-87 cancer cell lines as well as primary human breast cells were from American Type Culture Collection (ATCC) (Manassas, VA). Dermal human fibroblasts were obtained by written consent from two donors (approved by the Institutional Review Board at Sheba Medical Center, protocol # 7044). MDA-MB-231, MCF-7, BT-549 and U-87 were authenticated by short tandem repeat profiling.

### Growth conditions

MCF-7 cells were grown in low glucose Dulbecco modified eagle medium (DMEM), U87 cells were grown in high glucose DMEM, MDA-MB-231 and BT-549 cells in Roswell Park Memorial Institute (RPMI) 1640. Cell line media were supplemented with 10% fetal bovine serum (FBS), penicillin and streptomycin from Biological Industries Kibbutz Beit-Haemek, Il. Growth medium of BT-549 was also supplemented with 0.023 IU/ml of human recombinant insulin (Sigma, St. Louis, MO.). Fibroblasts were grown in high glucose DMEM supplemented with 20% FBS, penicillin-streptomycin, 1% non-essential amino acids and 0.2% β-mercaptoethanol (Invitrogen, Life Technologies Grand Island, NY). Primary normal human breast cells were grown in mammary epithelial cell basal medium containing recombinant human insulin, and TGFα, L-glutamine, epinephrine, apo-transferrin, pituitary extract and hydrocortisone hemisuccinate all from ATCC. For western blot analysis of cellular proteins cells were plated at a density of 4·10^3^ per cm^2^ and drugs were added 48 hours post-plating from stock solutions in dimethyl sulfoxide (DMSO) (Sigma, St. Louis MO). Controls received the vehicle. The concentration of DMSO in the growth medium did not exceed 0.08%.

### Drugs

Salubrinal was from Calbiochem-Merck4Biosciences (Darmstadt, Germany), vorinostat from LC laboratories (Boston, MA), ABT-888 from SelleckChem or APExBIO (Houston, TX), 6-TG from Tocris bioscience (Bristol, UK) or Sigma, (St. Louis, MO.), Z-VAD-FMK was purchased from APExBIO and senescence β-galactosidase staining kit was purchased from Cell Signaling Technologies (Boston, MA).

### Colony survival assay

Plating for colony survival assay, colony counting, and calculation of percent clonogenic death (CD) was performed in triplicates as described before [19–21]. Colonies were fixed, stained and counted 10–14 days following plating, when 90–95% of the colonies in the control possessed more than 50 cells. Experimental combination indices (CI) and simulated ones at ED50, ED75, ED90 and ED95 were obtained by employing the computer program CompuSyn according to Chou [21]. CI < 0.9 indicate synergistic interaction. Primary breast cells did not form colonies. The cells were therefore plated at a density of 300 cells/cm^2^ and the various drugs were added a day later for additional five days. Cell death was determined by trypan blue exclusion assay performed in triplicates and expressed as percent cell death (CD). The concentrations of drugs employed in our colony survival assay were at the range of plasma Cmax achieved in preclinical studies with laboratory animals [22,23] and in clinical studies with human [24].

### Western blot analysis

Preparation of cell lysates and analysis of treatment-induced changes in protein level and phosphorylation were done as we described before [19]. Protein content was determined with a bicinchoninic acid reagent (Bio-Rad, Hercules, CA), and equal loading was verified by measuring the absorbance at 520 nm of Ponceau S (Sigma, St-Louis, MO) extracted with phosphate buffer (2.67 mM KCl, 1.47 mM KH_2_PO_4_, 8.1 mM Na_2_HPO_4_ and 1.125 M NaCl) from individual strips of a twin run [19,20]. Blots were exposed to x-ray film for chemiluminescence following treatment with West Pico ECL reagent (Thermo Scientific Rockford, IL). Values for integrated light density of autoradiograms were obtained with Image J NIH software and were employed for determination of treatment-induced changes in protein levels and in the ratio of phosphorylated eIF2α (p-eIF2α) to the total level of the protein. Rabbit antibodies that recognize either p-eIF2α (#9721) or both (#9722) the phosphorylated and non-phosphorylated eIF2α, rabbit anti-RAD51 (#D4B10), rabbit anti-53BP1 (#4937), rabbit anti-PARP (#46D11) and rabbit monoclonal antibodies against c-IAP1, c-IAP2, survivin and XIAP (#9770) were from Cell Signaling Technologies (Boston, MA). Mouse monoclonal antibodies against BRCA1 (clone D9 #sc-6954) and against thioredoxin interacting protein (TXNIP) #sc-166234 were from Santa Cruz Biotechnology Inc. (Dallas, TX). Mouse monoclonal antibodies against Poly(ADP)ribose (PADPR) (10H #ab14459) were from abcamBiochemicals^®^ (Cambridge, UK).

### Senescence-associated β-galactosidase staining

Senescence β-galactosidase staining kit was from Cell Signaling Technologies and staining was performed according to the manufacturer instructions.

### Transfection of cells with plasmids coding for eIF2α variants

heIF2α S51A and heIF2α S51D in pcDNA3.CD2 were obtained from Addgene (Cambridge, MA). Transient transfection was performed with JetPei (Polyplus, New York, NY) according to manufacturer's instructions as we described before [19]. Experiments were carried out in triplicates in 6 well dishes, with 1.5 μg plasmid in 2 ml growth medium without antibiotics for 24 hours before adding 0.5 ml medium and proceeding with the experimental protocol.

### Tumor growth in nude mice

All animal studies were conducted according to experimental protocol # 609/10/ANIM which was approved by the Institutional Animal Care and Use Committee at Sheba Medical Center. Cells were suspended in a mixture of matrigel (reduced growth factor) (BD Biosciences, Bedford, MA) and RPMI (1:1). The cells (1.5*10^6^ in 200 μl) were injected into the inguinal mammary fat pad of athymic *nu*^-^/*nu*^-^ female mice (Harlan, Israel) weighing ~20 gr. Animals were weighed 1–2 times weekly and tumor size was measured 2–3 times weekly with a digital caliper. Tumor volume was estimated using the formula *V* = (*a* × *b*^2^) × 0.5, where *'a*' is the larger dimension and *'b*' the perpendicular diameter. Experiments included 7 mice per experimental group when testing the combined effect of vorinostat and 6-TG on tumor growth and 10 mice per group when testing the combined effect of vorinostat and ABT-888. The vehicle was composed of 40% PEG and 20% DMSO in 0.72% NaCl. Drugs were administrated daily through intraperitoneal injection (i.p.) throughout the experiment starting one or two days prior to cell inoculation and controls received the vehicle. The injection volume was 250 μl. Due to accidental damage caused by the injections, a few mice developed, within 24 hours post-injection, very large hematomas at the injected side that were associated with signs of distress (hunched posture and difficulties in movements). These mice were euthanized by CO_2_ asphyxiation. Their number in the experiment that tested the effect of combined vorinostat and ABT-888 treatment was as follows: C- (3 out of 11), V- (2 out of 10), ABT-888 –(none), V and ABT-888 (2 out of 11). The number of mice that died in the same fashion during the experiment that tested the effect of combined vorinostat and 6-TG treatment were as follows: C- (none) V- (1 out of 7) 6-TG—(1 out of 7) V and 6-TG—(1 out of 7). Experiments were ended about four weeks post-initiation when tumors started to show signs of necrosis. Mice were sacrificed by CO_2_ asphyxiation and blood samples were taken by cardiac puncture. Complete blood count along with liver and kidney functions were performed by A.M.L Veterinary Division, Herzlia, Il. Samples from internal organs were fixed in 4% buffered formalin and processed for hematoxylin and eosin (H&E) and Prussian blue staining.

### Statistical analysis

In vitro assays for clonogenic survival: Significance of differences between experimental groups receiving treatment with two agents and each of the groups receiving treatment with one agent or controls was verified with unpaired Student *t* test. *P* < 0.05 was considered statistically significant.

Experiments with nude mice: ANOVA with repeated measures was employed to test for the significance of the observed differences in tumor volume among the various experimental groups. The analysis included mice that survived throughout the experiments. Time within subject factor and V and ABT or V and 6-TG between subject factors were used. To approach normal distribution, the data were analyzed after square root transformation. ANOVA with repeated measures was also employed to demonstrate the lack of treatment-induced changes in mice weight. *p*< 0.05 was considered statistically significant.

## Results

### Vorinostat sensitizes cancer cells in vitro to treatment with ABT- 888

It has been reported that vorinostat reduces the level of DNA repair proteins in cancer but not in normal cells [8]. We too observed that vorinostat led to a dose-dependent decrease of BRCA1 and RAD51 level in MDA-MB-231 cells (S1 Fig). These results prompted us to test the effect of vorinostat on the sensitivity of cancer cells to the PARPi—ABT-888.

**Fig 1:**
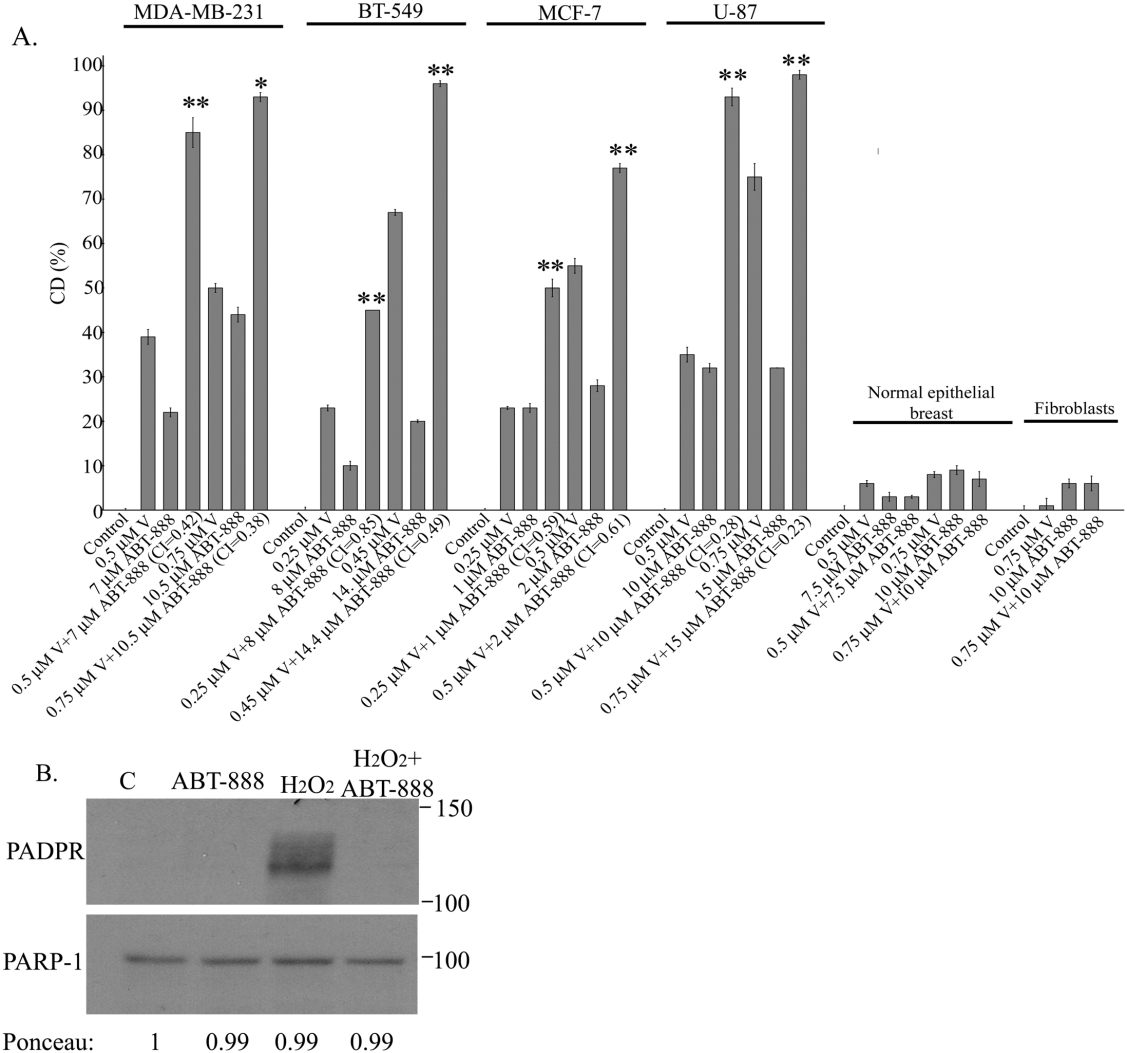
Vorinostat enhances the response of human cancer cell lines to ABT-888. **a**. Clonogenic death in response to combined treatment of vorinostat and ABT-888: Cancer cell lines and fibroblasts were plated for clonogenic survival assays and treated with vorinostat, ABT-888 or both. Values are means clonogenic death (CD) (%) of triplicates ± SEM. Determination of percent cell death (CD) for normal breast cells was performed by trypan blue exclusion assay. The experiment was reproduced once with similar results. Differences between CD of combined treatment and each of the sole treatments or the controls were significant. *p<0.05, **p<0.01. Combination indices (CI) were < 0.9. **b**. **ABT-888 is active within the cells**: MDA-MB-231 cells were incubated overnight with 10 μM ABT-888 than 15 min with 1 mM H2O2 before harvesting and processing for detection of poly (ADP-ribose) (PADPR) by western blot.

It has been reported that 5 μM ABT-888 leads to ~90% reduction in the clonogenic survival of MDA-MB-436 BRCA1 mutated breast cancer cell line [25]. In contrast, MDA-MB-231 which possesses wild type BRCA1 [26] responded poorly to treatment with 7–10 μM ABT-888 (Fig 1). Still, the inhibitor was active within the cells as demonstrated by its ability to abrogate H_2_O_2_-induced formation of poly(ADP)-ribose (Fig 1b). The triple negative BT-549 breast cancer cell line and the glioblastoma U-87 were similarly resilient to ABT-888 while the estrogen dependent MCF-7 breast cancer cell line was relatively sensitive to the drug. Nonetheless, as noted in Fig 1a, vorinostat-enhanced the response of all these cell lines to ABT-888 and all experimental and simulated CI indicated that the interaction between vorinostat and ABT-888 is synergistic (Fig 1a and S1 Table). Under our experimental conditions, normal human fibroblasts and breast cells were barely affected by any of these agents or their combination.

### Molecular mechanisms that underlie the sensitizing effect of vorinostat

**Fig 2:**
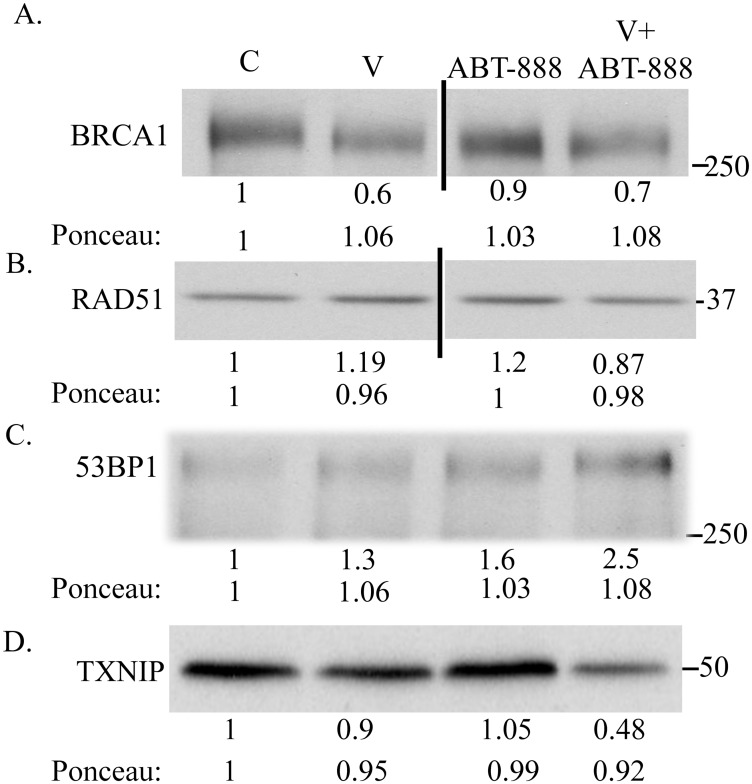
The effect of vorinostat, ABT-888 and their combination on cellular level of BRCA1, RAD51 and TXNIP. Combined treatment of vorinostat and ABT-888 reduced the level of BRCA1 by ~ 30% (a), did not affect the level of RAD51 (b), increased the level of 53BP1 relative to control ABT-888 or vorinostat treated cells (c) and decreased the level of TXNIP (d). ABT-888 (10 μM), vorinostat (0.5 μM) and their combination were added to the cells 48 hours post-plating. The cells were harvested 48 hours later and processed for western blot analysis of changes in the levels of BRCA1, RAD51 and TXNIP. Numbers at the bottom of the autoradiograms indicate changes relative to controls in the level of BRCA1, RAD51, TXNIP and of loaded proteins (Ponceau). The experiment was reproduced once with similar results. Unabridged images of the autoradiograms presented in panels (a) and (b) is shown in S2 Fig.

Using the experimental conditions depicted in Fig 1a we determined the level of DNA repair protein in cells treated with vorinostat, ABT-888 and their combination. In contrast to our expectations, we found that the combination of vorinostat and ABT-888 had only a moderate effect on the level of BRCA1, reducing it by no more than ~40%, while the level of RAD51 was unaffected. Intriguingly, the level of 53-BP1 was increased by vorinostat, and by ABT-888, and their combination increased the level of 53BP1 more than each drug alone (Fig 2a–2c). The possible implication of increased level of 53BP1 in the face of decreased level of BRCA1 for cellular sensitivity to PARPis is considered in the discussion (Fig 2).

In the absence of a dramatic effect of the combined treatment on the level of BRCA1 or RAD51 we determined the effect of vorinostat, ABT-888 and their combination on the level of cellular thioredoxin interacting protein (TXNIP). It has been reported before that increased expression of TXNIP underlies the differential effect of vorinostat on cancer cells [27]. We too demonstrated that high concentrations of vorinostat led to a dose dependent increase of TXNIP in MDA-MB-231 cells (S1 Fig). However, under our experimental conditions the combination of vorinostat and ABT-888 which enhanced cell death repeatedly led to a decrease rather than an increase in the level of TXNIP in MDA-MB-231 cells (Fig 2d).

**Fig 3:**
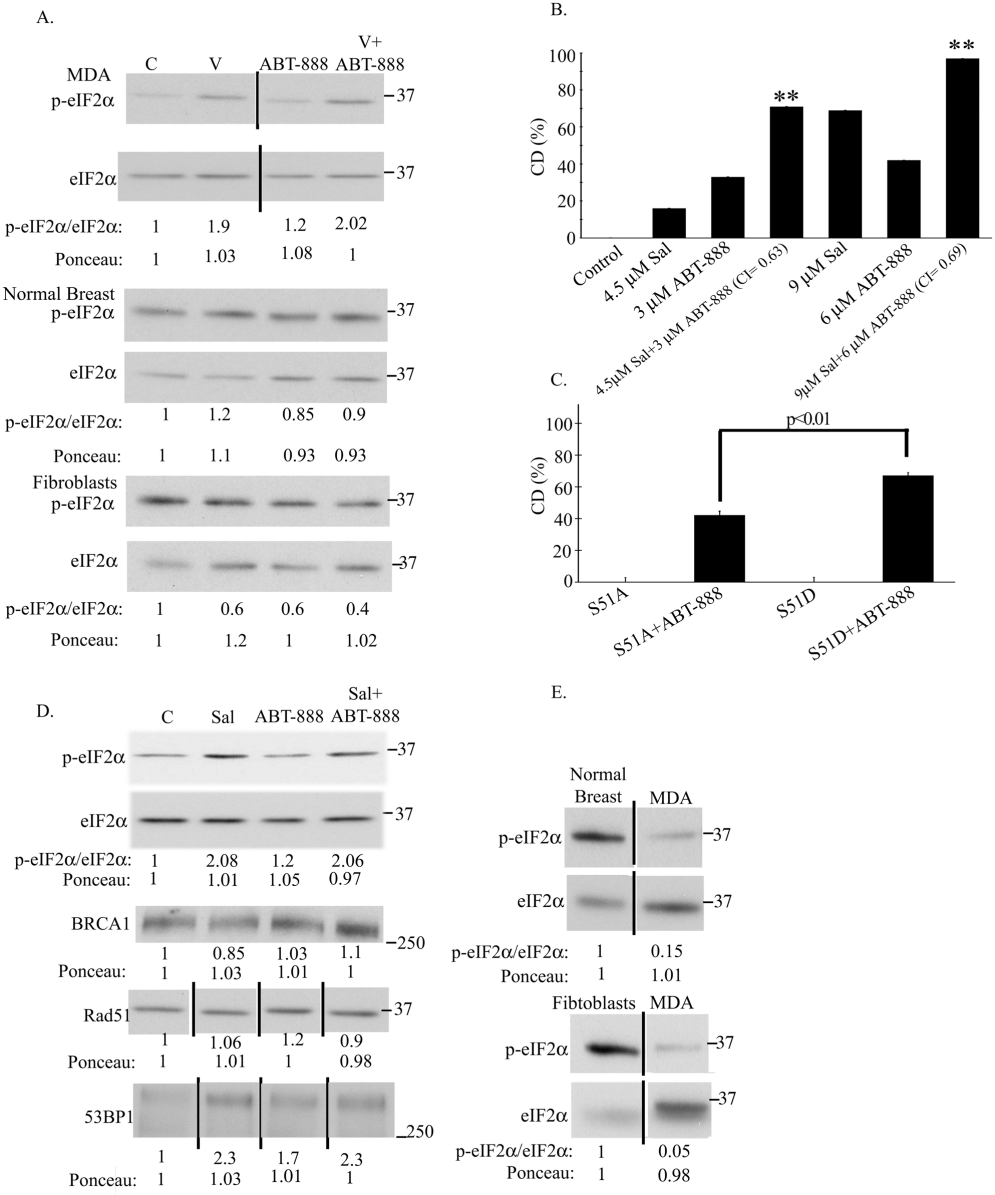
The effect of vorinostat on the sensitivity of cancer cells to ABT-888 is mediated by increased phosphorylation of eIF2α. a. Vorinostat increased eIF2α phosphorylation in ABT-888 treated MDA-MB-231 cells but not in ABT-888 treated fibroblasts and normal breast cells: ABT-888 (10 μM), vorinostat (0.5 μM) and their combination were added to the cells 48 hours post-plating. The cells were harvested 48 hours later and processed for western blot analysis of changes in the ratio p-eIF2α/eIF2α. Numbers at the bottom of the autoradiograms indicate changes relative to controls of p-eIF2α/ eIF2α and of loaded proteins (Ponceau). The experiment was reproduced once with similar results. b. Salubrinal increases CD of MDA-MB-231 cells in response to ABT-888: Cells were treated with salubrinal, ABT-888 or both. Values are means CD (%) of triplicates ± SEM. The experiment was reproduced once with similar results. Differences between CD (%) of combined treatment and each of the experimental treatments or controls were significant. **p<0.01, * *p*<0.05. CI were < 0.9. c. A phosphomimetic eIF2α variant increases clonogenic death in response to ABT-888: The PARPi (10 μM) was added to the cells 18 hours following transfection with 1.5μg/2ml eIF2α S51A or eIF2α S51D. Colonies were processed for analysis 10 days post-treatment initiation. Values are means CD (%) of triplicate samples ± SEM. The experiment was reproduced twice with similar results. Differences between each of the controls and their ABT-888 treated counterparts as well as between the ABT-888 treated S51A and S51D variants were significant. SA—non-phosphorylatable S51A eIF2α, SD—phosphomimetic S51D eIF2α. d. Salubrinal increases eIF2α phosphorylation and 53BP1 expression in ABT-888 treated cells without affecting the level of BRCA1 or RAD51: ABT-888 (10 μM) and salubrinal (4.5 μM) were added to the cells 48 hours post-plating. The cells were harvested 48 hours later and processed for western blot analysis of changes in BRCA1, RAD51, 53BP1 and the ratio p-eIF2α/eIF2α. Numbers at the bottom of the autoradiograms are changes relative to controls of the level of BRCA1, RAD51, 53P1, the ratio p-eIF2α/eIF2α and the amounts of loaded proteins (Ponceau). The experiment was reproduced once with similar results. e. The level of phosphorylated eIF2α is higher and the level of total eIF2α is lower in normal fibroblasts and breast cells than in cancer cells: Cells were harvested for western blot analysis four days post-plating. The experiment was reproduced once with similar results. Numbers at the bottom of the autoradiograms indicate changes relative to control of peIF2α/eIF2α and the amount of loaded proteins (Ponceau). The experiment was reproduced once with similar results. Unabridged images of the MDA autoradiogram in panel (a) of RAD51 and 53BP1 in panel (d) and of the autoradiograms in panel (e) are presented in S3 Fig.

In addition to TXNIP, increased phosphorylation of eIF2α has also been shown, by several laboratories including ours, to mediate the cytotoxic effect of vorinostat on cancer cells [19,28]. We therefore examined the level of eIF2α phosphorylation in cells treated with both vorinostat and ABT-888 and found that vorinostat at concentrations that enhanced sensitivity to ABT-888, led to a marked increase in eIF2α phosphorylation, while ABT-888 alone led to only a slight increase in the phosphorylation of the protein. In cells treated with both agents the level of phosphorylated eIF2α was similar to that obtained with vorinostat alone. In contrast, the combined action of vorinostat and ABT-888 failed to increase the level of phosphorylated eIF2α in human fibroblasts or in human normal breast cells (Fig 3a).

To determine if increased phosphorylation of eIF2α in ABT-888 treated cancer cells contributes to the sensitizing effect of vorinostat, we evaluated the effect of salubrinal—an inhibitor of eIF2α dephosphorylation—on the sensitivity of the cells to ABT-888. As noted in Fig 3b and in S2 Table, salubrinal enhanced the sensitivity of the cells to ABT-888, and similar results were obtained with the phosphomimetic eIF2α variant S51D (Fig 3c). In agreement with its effect on the sensitivity of the cancer cells to ABT-888, salubrinal mimicked the effect of vorinostat on increased phosphorylation of eIF2α in ABT-888 treated cells and similar to the combination of vorinostat and ABT-888, the combination of salubrinal and ABT-888 did not reduce the level of RAD51. However, in contrast to the effect of vorinostat, the sensitizing effect of salubrinal was achieved without at all affecting the level of BRCA1 (Fig 3d). Interestingly, the effect of salubrinal alone on the level of 53BP1 was higher than that seen with vorinostat or ABT-888 alone, and in cells treated with both salubrinal and ABT-888 the level of 53BP1 was higher than that observed in ABT-888 treated cells and similar to that noted in cells treated by salubrinal alone (Fig 3d).

Of note, in untreated fibroblasts or normal breast cells, the level of total eIF2α was much lower and the level of the phosphorylated protein was much higher than in the untreated human breast cancer cell line MDA-MB-231 (Fig 3e), suggesting that normal cells may be more tolerant than cancer cells to increased phosphorylation of eIF2α.

Under our experimental conditions, the sensitizing effect of vorinostat on the cellular response to ABT-888 was not affected by the presence of the pan-caspase inhibitor—z-vad-fmk (S4 Fig). A caspase-independent effect of vorinostat on cell survival has been demonstrated before by Xu et al. [29]. Curiously, z-vad-fmk by itself led to a dose-dependent decrease in cell survival—a phenomenon that has been observed before in various cellular systems [30–32].

Relatively high concentrations of vorinostat (5–10 folds higher than those employed in our studies) have been shown to dramatically reduce the level of the inhibitor of apoptosis protein (IAP) family such as c-IAP1, c-IAP2, XIAP and survivin [29]. We therefore examined the effect of vorinostat and that of the combined treatment of vorinostat and ABT-888 on the level of these proteins (S5 Fig). Under our experimental conditions, vorinostat did not affect the level of c-IAP1, survivin and XIAP. Also, ABT-888 did not affect the level of these proteins. The level of c-IAP2 was reduced by vorinostat treatment but was increased by ABT-888. Nonetheless, under our experimental conditions, the combined treatment did not to affect the level of these IAP.

The combined treatment of vorinostat and ABT-888 was however associated with an increased appearance of enlarged flat senescent looking cells which stained for β-galactosidase activity (S6 Fig). Here too, a similar effect of vorinostat has been reported before by Xu et al. [33].

Interestingly, similar to the treatment with vorinostat and ABT-888, treatment with salubrinal and ABT-888 led to an increased appearance of senescent looking cells which stained for β-galactosidase (S6 Fig). Also, like vorinostat, salubrinal did not affect the level of c-IAP1, survivin and XIAP but led to a decreased level of c-IAP2, and like vorinostat and ABT-888, combined treatment of salubrinal and ABT-888 did not affect the level of these IAP (S5 Fig).

### Increased inhibition of tumor xenograft growth in mice treated with both vorinostat and ABT-888

**Fig 4:**
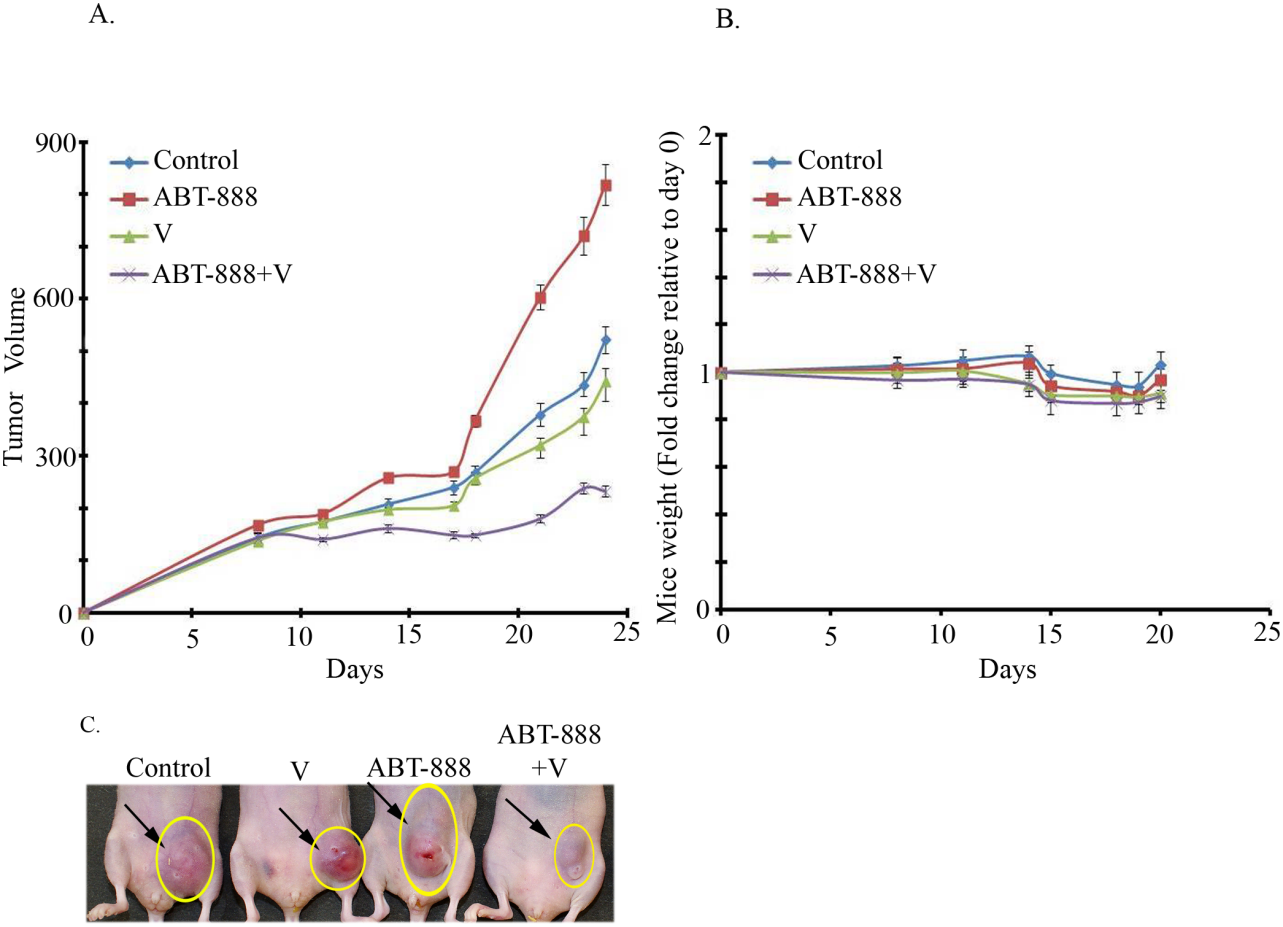
Combined treatment of Vorinostat and ABT significantly increases the inhibition of MDA-MB-231 tumor xenograft growth relative to each agent alone. a. Effect of the drug combination on tumor development: Differences between the various treatment groups were not detected at first measurements but developed over time. Administration of drugs was initiated two days prior to the injection of the cells on day zero. The combined treatment of vorinostat (200 mg/kg) and ABT-888 (40 mg/kg) had a significant (*p*<0.02) higher inhibitory effect on the development of tumor growth relative to each agent alone. Values are tumor volumes in mm^3^ ± SEM. b. Effect of treatments on mice weight: Mice were weighed throughout the experiments. The various drugs did not lead to significant change in weight. c. Samples of mice bearing tumors on last day of the experiment.

The effect of vorinostat on the sensitivity of MDA-MB-231 to ABT-888 was reproduced in vivo. Combined treatment of vorinostat and ABT-888 led to a significant inhibition of tumor growth relative to each treatment alone (Fig 4a). The various treatments did not lead to a significant effect on body weight (Fig 4b) and did not seem to affect complete blood count, liver or kidney functions (S3 and S4 Tables). H&E staining of sections from internal organ did not show treatment-induced changes in the heart, liver, kidney and lungs. Interestingly, brown staining was observed in the spleen from vorinostat or vorinostat and ABT-888 treated mice (panel 'a' in S7 Fig). It has been noted before that accumulation of hemosiderin in the spleen of cisplatin-treated mice was associated with brown staining in H&E stained preparations [34]. Indeed Prussian blue staining revealed an increased level of splenic iron in vorinostat or vorinostat and ABT-888 treated mice (panel 'b' in S7 Fig). The reason for this is unknown as yet. Although vorinostat did not seem to cause anemia or decreased level of hemoglobin in the mice it may affect iron metabolism through other means such as increased expression of ferritin H [35] and frataxin [36]. Interestingly, although vorinostat has been approved for cancer treatment, its effect on accumulation of iron deposit in the spleen has not been reported before.

### Vorinostat sensitizes cancer cells in vitro to treatment with 6-TG and significantly increases inhibition of tumor xenograft growth relative to each agent alone

**Fig 5:**
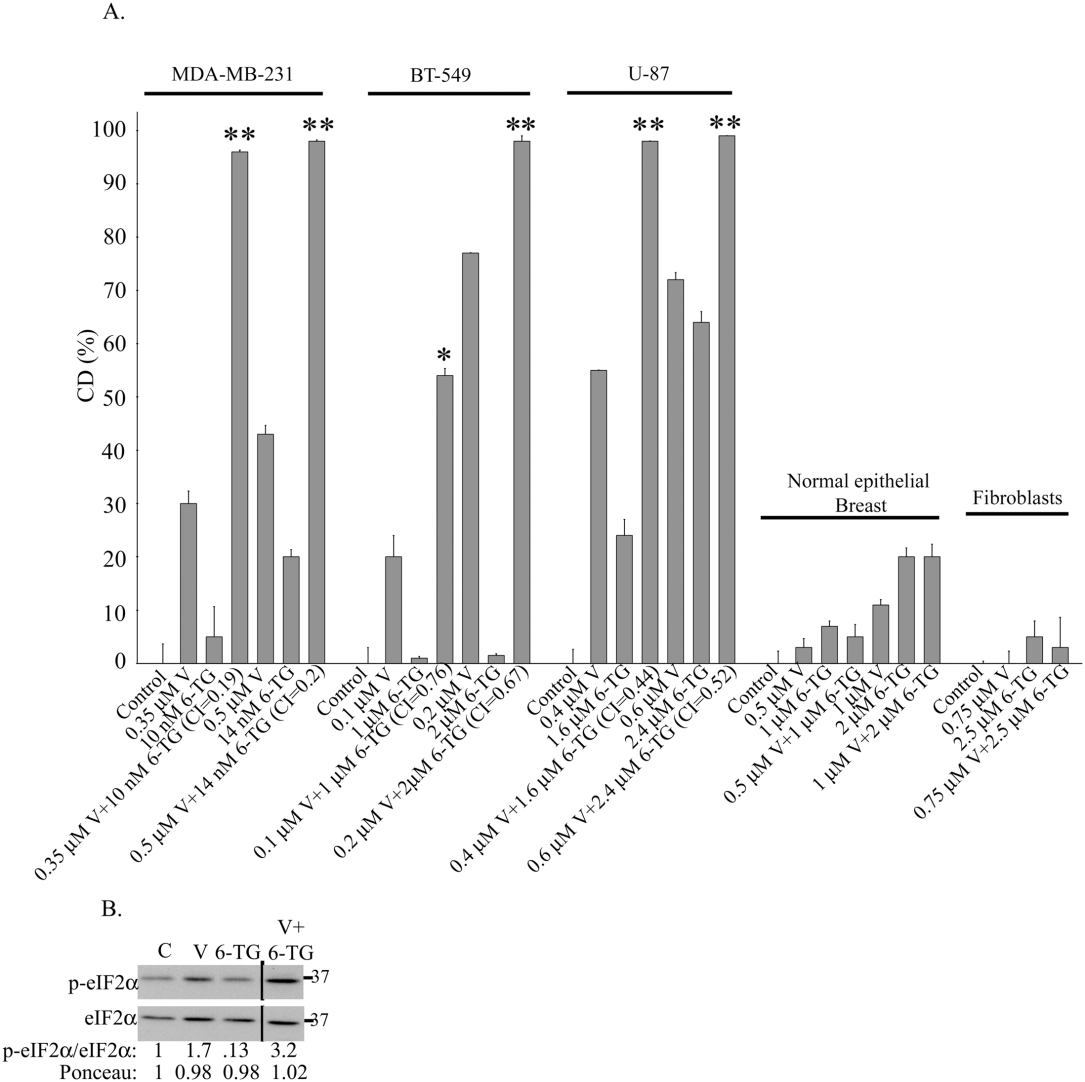
Combined treatment of vorinostat and 6-TG enhances clonogenic cancer cell death and increases phosphorylation of eIF2α. a. Combined treatment of vorinostat and 6-TG led to enhanced clonogenic cell death in cancer cells. Cells plated for clonogenic survival assays were treated with vorinostat, 6-TG or both. Values are means CD (%) of triplicates ± SEM. Determination of percent cell death (CD) for normal breast cells was performed by trypan blue exclusion assay. Differences between CD (%) of combined treatment and each of the treatments or controls were significant **p<*0*.*01*, *p<0.05. The experiments were reproduced once with similar results. CI values were < 0.9. b. Combined treatment of vorinostat and 6-TG increased phosphorylation of eIF2α: 6-TG (10 nM) and vorinostat (0.5 μM) were added to MDA-MB-231 cells 48 hours post-plating. The cells were harvested 48 hours later and processed for western blot analysis of changes in the ratio p-eIF2α/eIF2α. Numbers at the bottom of the autoradiograms indicate changes relative to controls of p-eIF2α/ eIF2α and of the loaded proteins (Ponceau). The experiment was reproduced once with similar results. An unabridged image of the autoradiogram in presented in S8 Fig.

In addition to its effect on the sensitivity to ABT-888, vorinostat enhanced the sensitivity of cancer cells to 6-TG (Fig 5, S5 Table). As mentioned above 6-TG is known to target cells that are sensitive to PARPis. However, although MDA-MB-231 were resistant to ABT-888, nM of 6-TG in combination with vorinostat were sufficient to produce a synergistic effect which was associated with a marked increase in the phosphorylation of eIF2α (Fig 5b). In contrast, other cell lines required μM levels of 6-TG in combination with vorinostat to produce the enhanced response. Human normal fibroblasts and breast cells were not affected by any of these agents or their combination.

**Fig 6:**
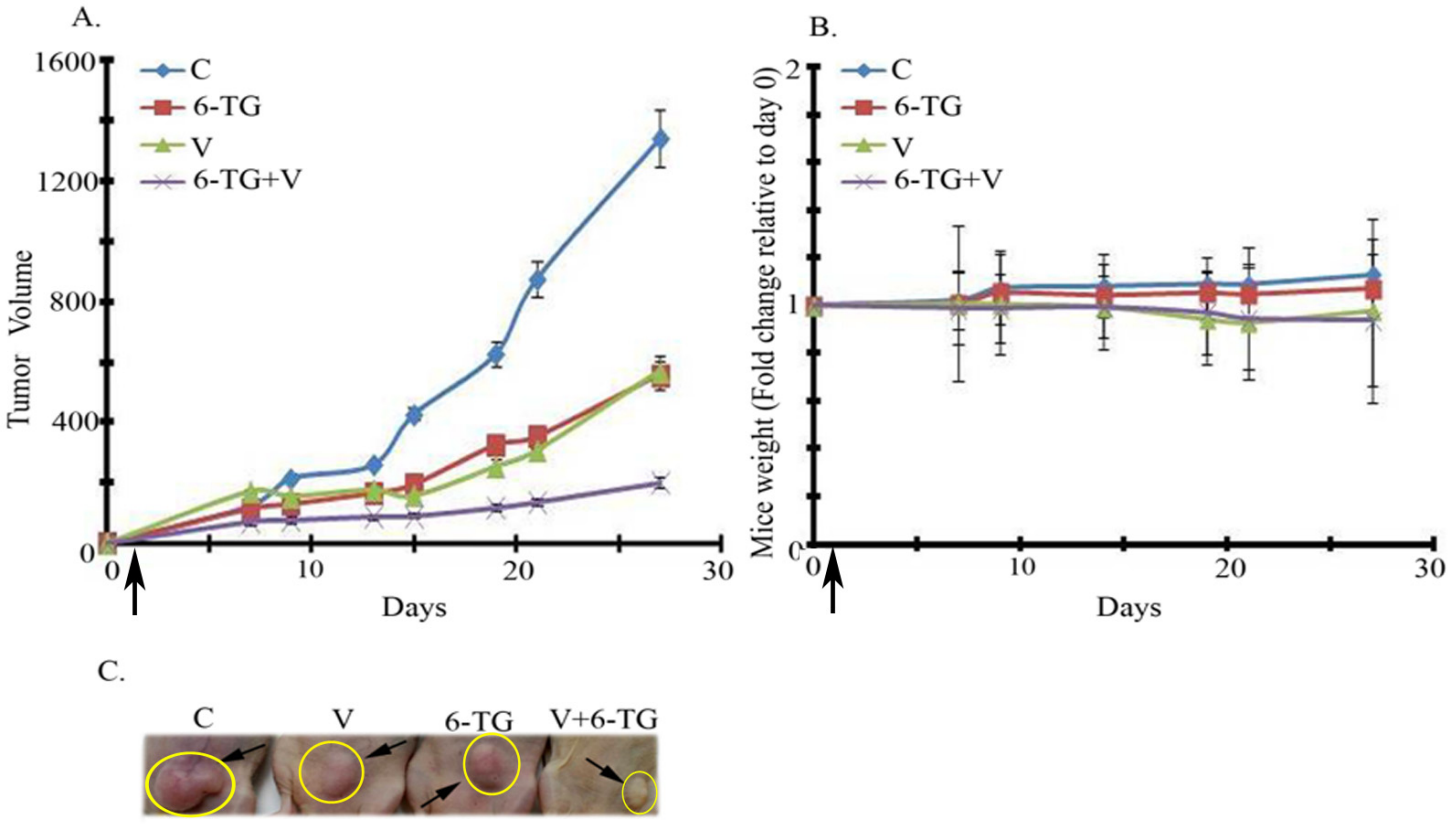
Combined treatment of Vorinostat and 6-TG significantly increases the inhibition of MDA-MB-231 tumor xenograft growth relative to each agent alone. a. Effect of the drug combination on tumor development: Differences between the various treatment groups were not detected at first measurements but developed over time. Administration of drugs was initiated one day prior to the injection of the cells on day zero. The combined treatment of vorinostat (150 mg/kg) and 6-TG (0.75 mg/kg) had a significant (*p*<0.03) inhibitory effect on the development of tumor growth relative to each agent alone. Values are tumor volumes in mm^3^ ± SEM. b. Effect of treatments on mice weight: Mice were weighed throughout the experiments. The various drugs did not lead to significant change in weight. c. Samples of mice bearing tumors on last day of the experiment.

Combined treatment of vorinostat and 6-TG led to a marked increase in the inhibition of MDA-MB-231 tumor xenograft growth relative to each agent alone. The various treatments did not lead to significant weight loss (Fig 6). 6-TG alone or in combination with vorinostat led to occasional mild leucopenia and mild to moderate anemia which are known and treatable side effects of anti-neoplastic treatments (S6 Table). Neither 6-TG nor its combination with vorinostat influenced kidney or liver functions (S7 Table). H&E staining of sections from internal organ of control or vorinostat and 6-TG treated mice did not show treatment-induced changes in the heart, liver, kidney and lungs. However, similar to the spleens in vorinostat or vorinostat and ABT-888 treated mice, brown staining was observed in the spleens of mice treated with the combination of vorinostat and 6-TG (S9 Fig). In this case, the brown staining may reflect a treatment-dependent decrease in red blood cells.

## Discussion

The underlying hypothesis that prompted this study was that decreased level of DNA repair proteins will sensitize cells to PARPis, and since vorinostat has been demonstrated to specifically reduce the level of DNA repair proteins in cancer but not in normal cells [8] we chose to evaluate its effect on the sensitivity of cancer cells to ABT-888. Our initial results showed that micromolar concentrations of vorinostat could lead to a major reduction in the level of BRCA1 and RAD51 and as expected vorinostat led to increased sensitivity of various cancer cell lines to ABT-888. Most notable was its sensitizing effect on the triple negative MDA-MB-231 and BT-549 breast cancer cell lines and the glioblastoma U-87 cell line that showed poor response to ABT-888 when added alone. Intriguingly, the low concentrations of vorinostat employed in the clonogenic survival assay resulted in no more than ~ 40% reduction in the level of BRCA1 and had no effect on RAD51.

While this study was ongoing several laboratories showed that combining HDACis with specific PARP-1 inhibitors led to enhanced cell kill of cultured human cancer cell lines [10–14]. The increased effect on cell survival correlated with a partial decrease in the level of DNA repair proteins. Very recently, when this manuscript was in its final stages, Ha et al., showed that combining HDACis and PARPis led to decreased survival of triple negative breast cancer cells in vitro and delayed development of MDA-MB-231 tumor xenograft in vivo [12]. Here too the physiological effect was correlated with a partial decrease in the level of various DNA repair proteins.

While synthetic lethality to PARPis was originally associated with bi-allelic mutations in BRCA1/2 [2], it is possible that the enhanced response to ABT-888 seen in our experiments and in the above cited studies results from a cumulative effect of vorinostat on the level of various DNA repair proteins. Of note is our finding that combined treatment of vorinostat and ABT-888 leads to increased level of 53BP1. The competition between 53BP1 and BRCA1 for binding to DSBs is well documented, and deletion of 53BP1 abrogates the sensitivity of BRCA1 mutated cells to PARPis [6,37]. Hence, it is tempting to postulate that decreased level of BRCA1 combined with increased level of 53BP1 participate in mediating the enhanced clonogenic death seen in response to treatment with vorinostat and ABT-888. However, it has been demonstrated that increased acetylation of H4K16 following treatment with HDACi favors the association of BRCA1 with chromatin and diminishes that of 53BP1 [38]. Therefore the exact role played by 53BP1 in mediating the enhanced response to ABT-888 in vorinostat-treated cells under our experimental conditions requires further analysis.

It is however clear that a thorough understanding of the interactions among the various components of the DNA repair machinery in each experimental system is required before pointing to a partial decrease in the level of specific DNA repair proteins as the cause for the sensitizing effect of vorinostat.

Several laboratories including ours showed that vorinostat leads to increased phosphorylation of eIF2α [19,28], which is known to affect the response of cancer cells to anti-neoplastic treatments including vorinostat [19,28,39,40]. We also showed that increased phosphorylation of eIF2α in MDA-MB-231 interferes with DNA repair [19]. By employing both salubrinal and the phosphomimetic eIF2α S51D variant, our experiments clearly demonstrate that increased eIF2α phosphorylation in and of itself sensitizes cancer cells to treatment with ABT-888. However, this does not exclude the possibility that in cells treated with both vorinostat and ABT-888 other downstream effectors of vorinostat are involved in the observed effect. It is therefore possible that vorinostat may sensitize cancer cells to ABT-888 by involving different combinations of molecular pathways than those activated by drugs that affect eIF2α phosphorylation.

Importantly, in contrast to the combined effect of vorinostat and ABT-888, combined treatment of salubrinal and ABT-888 did not lead to reduced level of BRCA1, stressing the point made above that observed changes in the level of specific DNA repair proteins may not fully explain the sensitizing effect of vorinostat. The level of 53BP1 in cells treated with both salubrinal and ABT-888 was higher than in cells treated with ABT-888 alone. Intriguingly, the level of 53BP1 in cells treated with salubrinal was higher than in cells treated with vorinostat alone. Here too, the role of 53BP1 in mediating the effect of salubrinal on the sensitivity of the cells to ABT-888 remains to be determined.

In addition to its sensitizing effect on cellular response to ABT-888, vorinostat also increased the sensitivity of cancer cells to 6-TG, and combined treatment of vorinostat and 6-TG increased the phosphorylation of eIF2α relative to each agent alone. In contrast, neither drug combinations affected the survival of normal human fibroblasts and breast cells.

Notably, the in vivo experiments with MDA-MB-231 tumor xenografts showed that the combined treatment of vorinostat and ABT-888 or vorinostat and 6-TG led to a significant increase in the inhibition of tumor growth relative to each drug alone. Our studies also addressed the effect of the combined treatments on the animals' weight, liver and kidney functions and complete blood count. In general, both treatments were well tolerated and did not affect liver and kidney functions nor did they cause significant weight loss or noted pathological changes in H&E stained sections of internal organs. The combination of 6-TG with vorinostat led to mild leucopenia and mild to moderate anemia which is reversible and potentially treatable.

In sum, our results suggest that cancer patients could benefit from the combination of ABT-888 and vorinostat regardless of their tumor's innate resistance to ABT-888 due to active HRR. The combination of vorinostat and 6-TG offers an additional treatment option for patients with innate or acquired resistance to PARPis due to secondary or reversal BRCA mutations, decreased PARP-1 level or increased MDR1 level in the cells. The drugs utilized in this project are either already FDA approved for patient use (vorinostat, 6-TG) or are in advanced stages of clinical testing (PARP inhibitors). Hence their inclusion in clinical trials may not take long.

The fact that together PARPis and HDACis directly affect chromatin structure, suggests that the combination of these inhibitors could circumvent resistance to other drugs that target upstream signal transduction components of growth factors and hormones. Thus, this combination may be active in other scenarios where cells have demonstrated resistance, e.g. Trastuzumab and lapatinib resistant HER2^+^ breast cancer, hormone resistant ER^+^ breast cancer, hormone resistant PR^+^ prostate cancer, as well as triple negative breast cancers.

It is however possible, that vorinostat-resistant cancers will not respond to treatment with vorinostat and PARPis or vorinostat and 6-TG. Thus, our finding regarding the effect of phosphorylated eIF2α on the sensitivity of cancer cells to PARPis may offer an additional treatment option for vorinostat resistant cancers. Also, because the level of phosphorylated eIF2α is much higher and that of the total amount of the proteins was much lower in normal fibroblasts and breast cells than in MDA-MB-231 cells, the growth and proliferation of normal cells may be more tolerant to increased eIF2α phosphorylation than MDA-MB-231, and drugs that directly to increase phosphorylation of eIF2α [19,39] may differentially affect cancer cells by providing a similar sensitizing effect to that of vorinostat without triggering all of its downstream effectors, thus minimizing potential side effect.

## Supporting Information

S1 Fig A dose-dependent effect of vorinostat on the level of BRCA1, RAD51 and TXNIP in MDA-MB-231 cells. Cells were harvested 48 hours following addition of vorinostat and processed for western blot analysis of BRCA1, RAD51 and TXNIP. Numbers at the bottom of each autoradiogram are changes relative to controls in the level of the specific proteins and in the amount of total loaded proteins (Ponceau). C- Control—Cells incubated with the vehicle. 0.5V –Cells incubated with 0.5 μM vorinostat. 2V –Cells incubated with 2 μM vorinostat.
(TIF)

S2 Fig Unabridged images of autoradiograms shown in Fig 2. (TIF)

S3 Fig Unabridged images of autoradiograms shown in Fig 3. (TIF)

S4 Fig Z-VAD-FMK does not affect survival of cells treated with both vorinostat and ABT-888. Values are means clonogenic death (CD) (%) of triplicates ± SEM. Differences between %CD of combined treatment and each of the sole treatments or the controls were significant **p<0.01. V- vorinostat—Cells incubated with 0.5 uM, ABT-888 –cell incubated with 10 uM of the inhibitor, z-vad-fmk—cells were incubated with 20 or 40 uM of the inhibitor as indicated. Control received the vehicle (DMSO).
(TIF)

S5 Fig Combined treatments of vorinostat and ABT-888, or vorinostat and salubrinal do not affect the level of c-IAP1, c-IAP2, survivin and XIAP. Cells were harvested 48 hours following addition of drugs and processed for western blot analysis of c-IAP1, c-IAP2, surivivin and XIAP. Numbers at the bottom of each autoradiogram are changes relative to controls in the level of the specific proteins and in the amount of total loaded proteins (Ponceau). C- Control—Cells incubated with the vehicle. V—Cells incubated with 0.5 μM vorinostat. ABT-888 –Cells incubated with 10 μM ABT-888, V+ABT-888 cells treated with both drugs, sal- salubrinal (4.5 μM) sal+ABT-888 –cells incubated with both drugs.
(TIF)

S6 Fig Combined treatments of vorinostat and ABT-888, or vorinostat and salubrinal is associated with increased appearance of senescence looking cells and staining for α-galactosidase activity. Cells were plated for colony survival assay and incubated with vorinostat (0.5 uM), ABT-888 (10 uM) vorinostat and ABT-888, salubrinal (4.5 uM) and salubrinal and ABT-888. Control received the vehicle. Cells were stained for senescence associated β-galactosidase activity (reflected in the blue stain). Numbers are average of senescent looking cells/colony ± SEM in triplicate plates and differences between the combined treatment and each one of the sole treatments or the control was statistically significant p<0.01.
(TIF)

S7 Fig The effect of vorinostat, ABT-888 and their combination on internal organs. a. Sections were stained with H&E: The arrows point to brown stained areas in the spleen. b. Sections stained with Prussian blue: Increased blue staining is noted in spleen from vorinostat and vorinostat and ABT-888 treated mice.
(TIF)

S8 Fig An unabridged image of autoradiogram shown in Fig 5. (TIF)

S9 Fig The effect of combined vorinostat and 6-TG treatment on internal organs. Sections were stained with H&E. The arrows point to brown stained areas in the spleen.
(TIF)

S1 Table Vorinostat enhanced the sensitivity of cancer cells to ABT-888. Cells plated for clonogenic survival assays were treated with vorinostat and ABT-888 at the specified ratios of vorinostat to ABT-888 as indicated in the table and in Fig 1. CI at effective doses of the drug combinations that leads to 50%, 75%, 90% and 95% clonogenic death were derived by employing the computer program CompuSyn. The low (< 0.9) CI values indicate synergistic interaction.
(PDF)

S2 Table Salubrinal enhanced the sensitivity of MDA-MB-231 breast cancer cells to ABT-888. Cells plated for clonogenic survival assays were treated with salubrinal and ABT-888 at the specified ratio as described in Fig 3B. CI at effective doses of the drug combinations that leads to 50%, 75%, 90% and 95% clonogenic death were derived by employing the computer program CompuSyn. The low (< 0.9) CI values indicate synergistic interaction.
(PDF)

S3 Table The effect of vorinostat, ABT-888 and their combination on blood hematocrit of treated mice. Mice were treated with 200 mg/kg vorisnotat, 40 mg/kg ABT-888 or their combinations. Controls received the vehicle. Blood was drawn by cardiac puncture from randomly sampled mice of each experimental group for determination of their hematocrit. * According to A.M.L. C-Control, V-vorinostat.
(PDF)

S4 Table The effect of vorinostat, ABT-888 and their combination on blood hematocrit of treated mice. Mice were treated with 200 mg/kg vorisnotat, 40 mg/kg ABT-888 or their combinations. Controls received the vehicle. Blood was drawn by cardiac puncture from randomly sampled mice of each experimental group for determination of their hematocrit. * According to A.M.L. C-Control, V-vorinostat.
(PDF)

S5 Table Vorinostat enhanced the sensitivity of cancer cells to 6-TG. Cells plated for clonogenic survival assays were treated with vorinostat and 6-TG at the specified ratio of as indicated in the table and in Fig 5. CI at effective doses of the drug combinations that leads to 50%, 75%, 90% and 95% clonogenic death were derived by employing the computer program CompuSyn. The low (< 0.9) CI values indicate synergistic interaction.
(PDF)

S6 Table The effect of vorinostat, 6-TG and their combination on blood hematocrit of treated mice. Mice were treated with 150 mg/kg vorinostat, 0.75 mg/kg 6-TG or their combinations. Control received the vehicle. Blood was drawn by cardiac puncture from randomly sampled mice of [29] each experimental group for determination of their hematocrit. *According to A.M.L. C-Control, V-vorinostat.
(PDF)

S7 Table The effect of vorinostat, 6-TG and their combination on blood chemistry. Blood was drawn by cardiac puncture from randomly sampled mice of each experimental group for determination of blood chemistry. * According to A.M.L. SGOT- Aspartate aminotransferase, SPGT—Alanine transaminase. C-Control, V-vorinostat (150 mg/kg). 6-TG (0.75 mg/kg).
(PDF)
