# Correction: Overcoming Resistance of Cancer Cells to PARP-1 Inhibitors with Three Different Drug Combinations

**DOI:** 10.1371/journal.pone.0161799

**Published:** 2016-08-22

**Authors:** Michal Yalon, Liron Tuval-Kochen, David Castel, Itai Moshe, Inbal Mazal, Osher Cohen, Camila Avivi, Kineret Rosenblatt, Sarit Aviel-Ronen, Ginette Schiby, Joachim Yahalom, Ninette Amariglio, Raphael Pfeffer, Yaacov R. Lawrence, Amos Toren, Gideon Rechavi, Shoshana Paglin

The fourteenth author’s name is spelled incorrectly. The correct name is: Yaacov R. Lawrence

Additionally, there are errors in the author affiliations. The affiliations should appear as shown here:

Michal Yalon^1,2^, Liron Tuval-Kochen^2,3^, David Castel^3,4^, Itai Moshe^1^, Inbal Mazal^2^, Osher Cohen^5^, Camila Avivi^6^, Kineret Rosenblatt^6^, Sarit Aviel-Ronen^6^, Ginette Schiby^6^, Joachim Yahalom^7^, Ninette Amariglio^2,8^, Raphael Pfeffer^2^, Yaacov R. Lawrence^2,3^, Amos Toren^1,2^, Gideon Rechavi^2,3^, Shoshana Paglin^2^

1 Department of Pediatric Hematology-Oncology, Safra Children's Hospital, Sheba Medical Center Ramat-Gan 52621, Israel

2 Cancer Research Center, Sheba Medical Center, Ramat-Gan 52621, Israel

3 Sackler School of Medicine, Tel Aviv University, Tel Aviv 69978, Israel

4 Neufeld Cardiac Research Institute, Sheba Medical Center, Ramat-Gan 52621, Israel

5 Department of Surgery, Sheba Medical Center, Ramat-Gan 52621, Israel

6 Department of Pathology, Sheba Medical Center, Ramat-Gan 52621, Israel

7 Department of Radiation Oncology, Memorial Sloan Kettering, NYC 10021, USA

8 The Mina and Everard Goodman Faculty of Life Sciences, Bar-Ilan University, Ramat-Gan 52900, Israel

Finally, there are errors in the funding section. The complete, correct Funding Statement is: http://ec.europa.eu/research/mariecurieactions/ Grant # PCIG10-GA-2011-303795 (Y.L.), Chaya and Kadish Shermeister endowment (The Laboratory of Experimental Radiotherapy), the estate of Miss HARAN ESTER of blessed memory through Keren Izvonot, Israel Ministry of Health (S.P.) and the Bencuya Family Foundation (L.T.K., R.P.). The funders had no role in study design, data collection and analysis, decision to publish, or preparation of the manuscript.
