# Supplementary figures and images for: Overcoming Resistance of Cancer Cells to PARP-1 Inhibitors with Three Different Drug Combinations
Source: PLoS One. 2016 May 19;11(5):e0155711. doi: 10.1371/journal.pone.0155711 (PMC4873128; doi:10.1371/journal.pone.0155711)

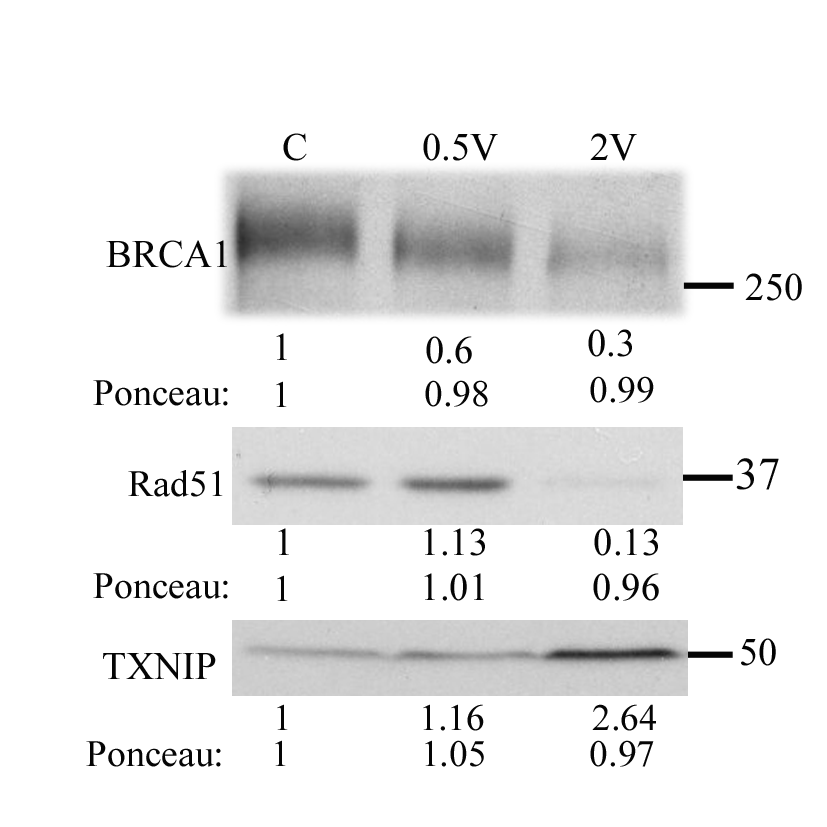

Supplement: S1 Fig — Cells were harvested 48 hours following addition of vorinostat and processed for western blot analysis of BRCA1, RAD51 and TXNIP. Numbers at the bottom of each autoradiogram are changes relative to controls in the level of the specific proteins and in the amount of total loaded proteins (Ponceau). C- Control—Cells incubated with the vehicle. 0.5V –Cells incubated with 0.5 μM vorinostat. 2V –Cells incubated with 2 μM vorinostat. (TIF) [file pone.0155711.s001.tif]

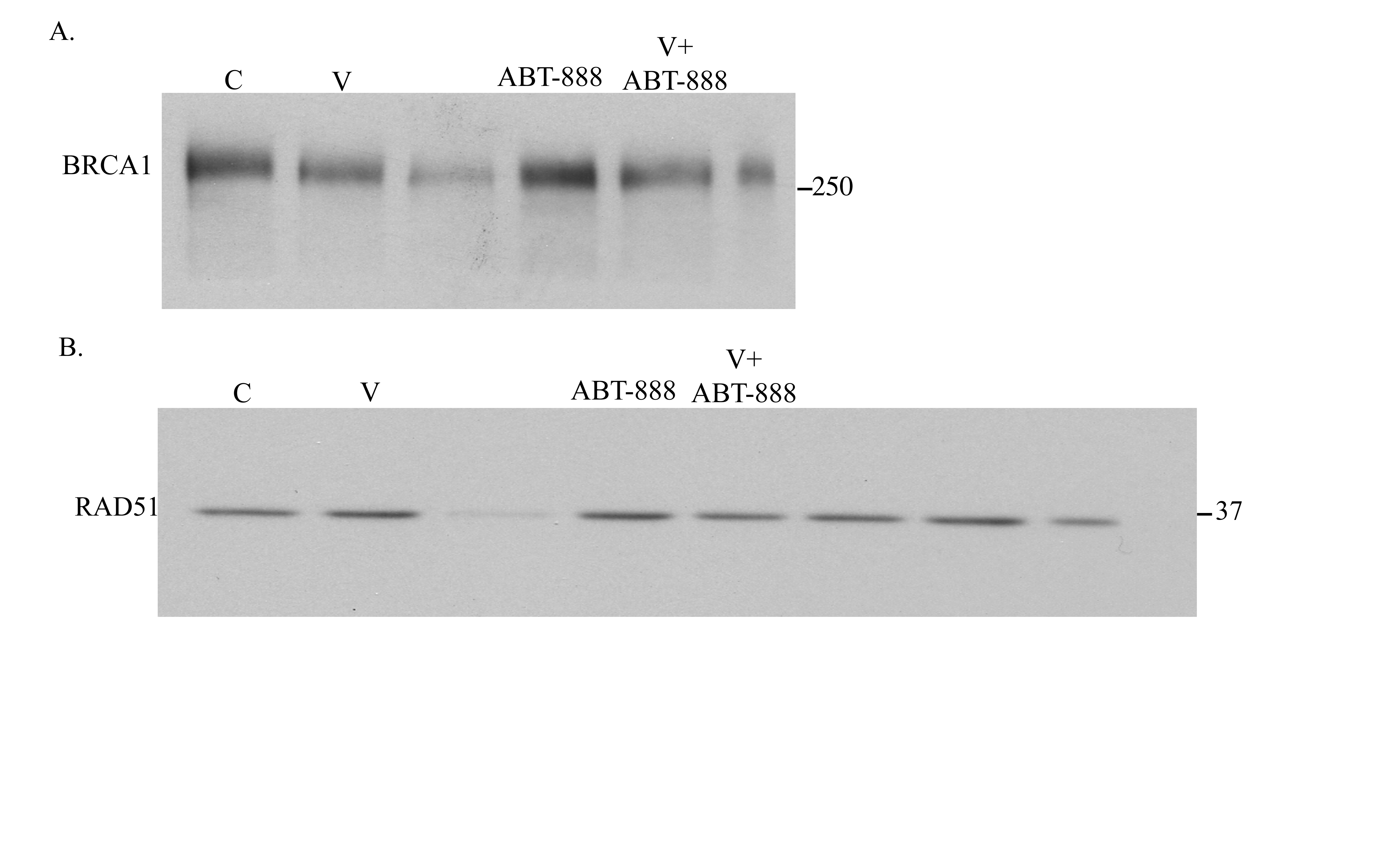

Supplement: S2 Fig — (TIF) [file pone.0155711.s002.tif]

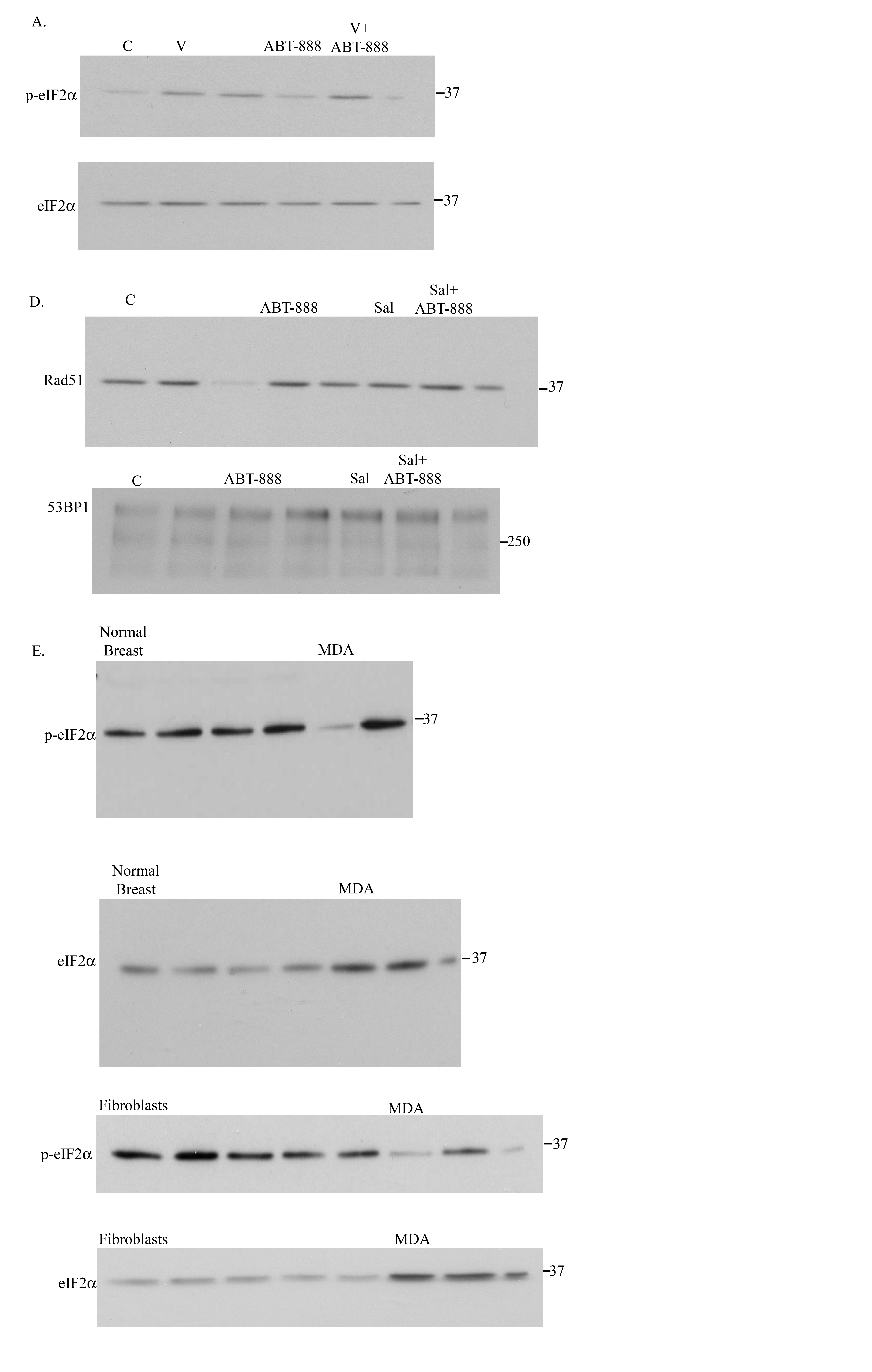

Supplement: S3 Fig — (TIF) [file pone.0155711.s003.tif]

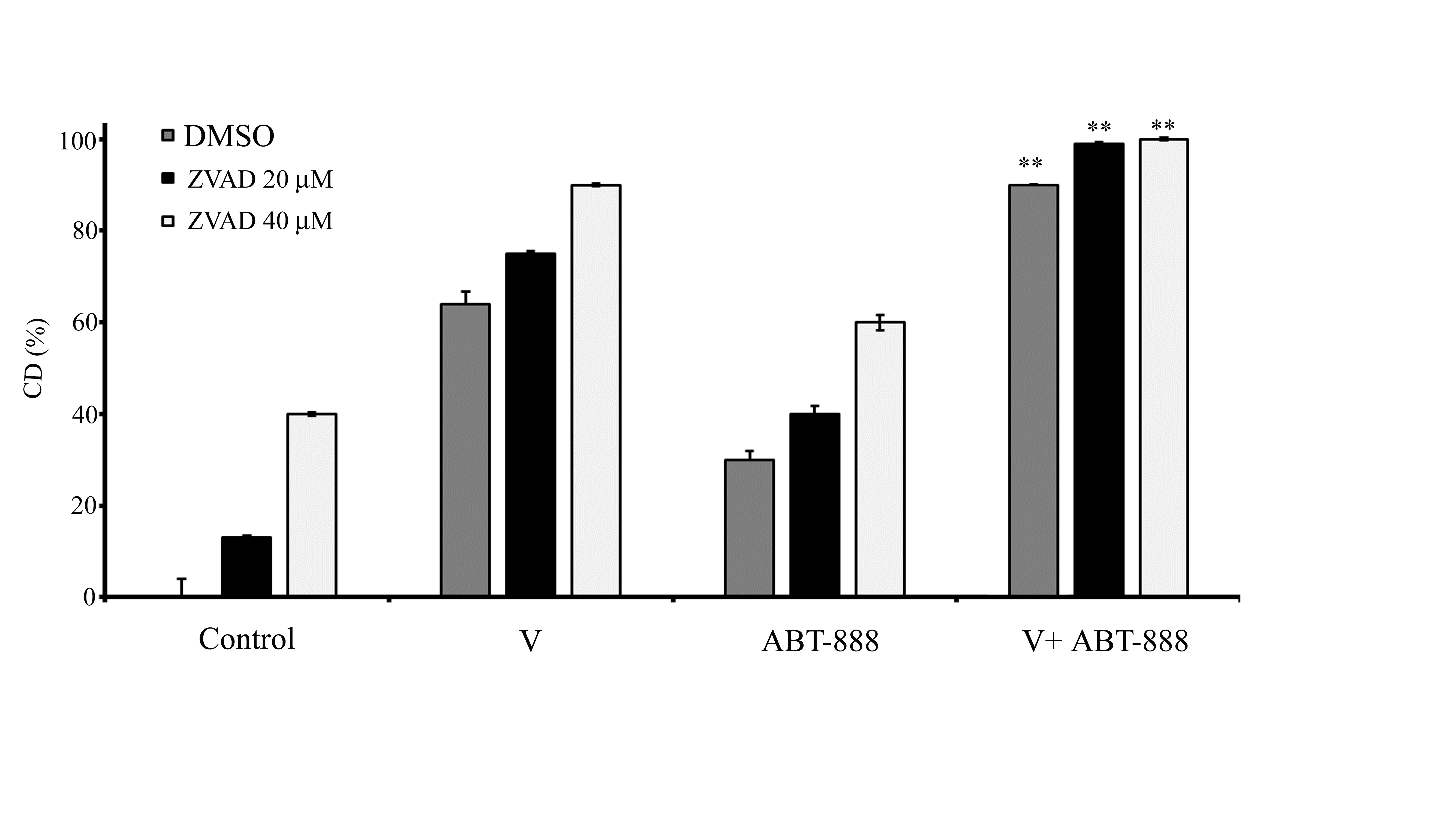

Supplement: S4 Fig — Values are means clonogenic death (CD) (%) of triplicates ± SEM. Differences between %CD of combined treatment and each of the sole treatments or the controls were significant **p<0.01. V- vorinostat—Cells incubated with 0.5 uM, ABT-888 –cell incubated with 10 uM of the inhibitor, z-vad-fmk—cells were incubated with 20 or 40 uM of the inhibitor as indicated. Control received the vehicle (DMSO). (TIF) [file pone.0155711.s004.tif]

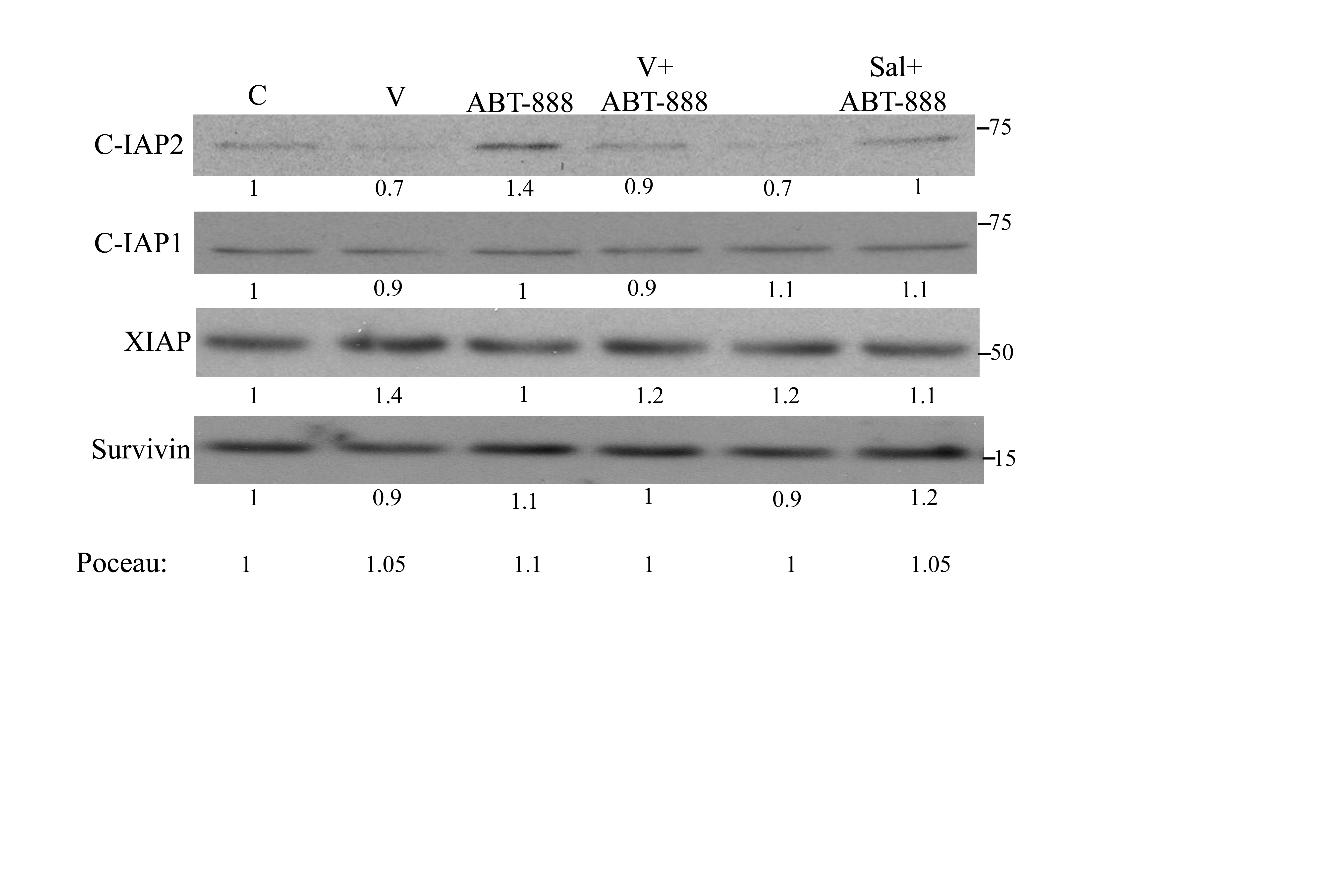

Supplement: S5 Fig — Cells were harvested 48 hours following addition of drugs and processed for western blot analysis of c-IAP1, c-IAP2, surivivin and XIAP. Numbers at the bottom of each autoradiogram are changes relative to controls in the level of the specific proteins and in the amount of total loaded proteins (Ponceau). C- Control—Cells incubated with the vehicle. V—Cells incubated with 0.5 μM vorinostat. ABT-888 –Cells incubated with 10 μM ABT-888, V+ABT-888 cells treated with both drugs, sal- salubrinal (4.5 μM) sal+ABT-888 –cells incubated with both drugs. (TIF) [file pone.0155711.s005.tif]

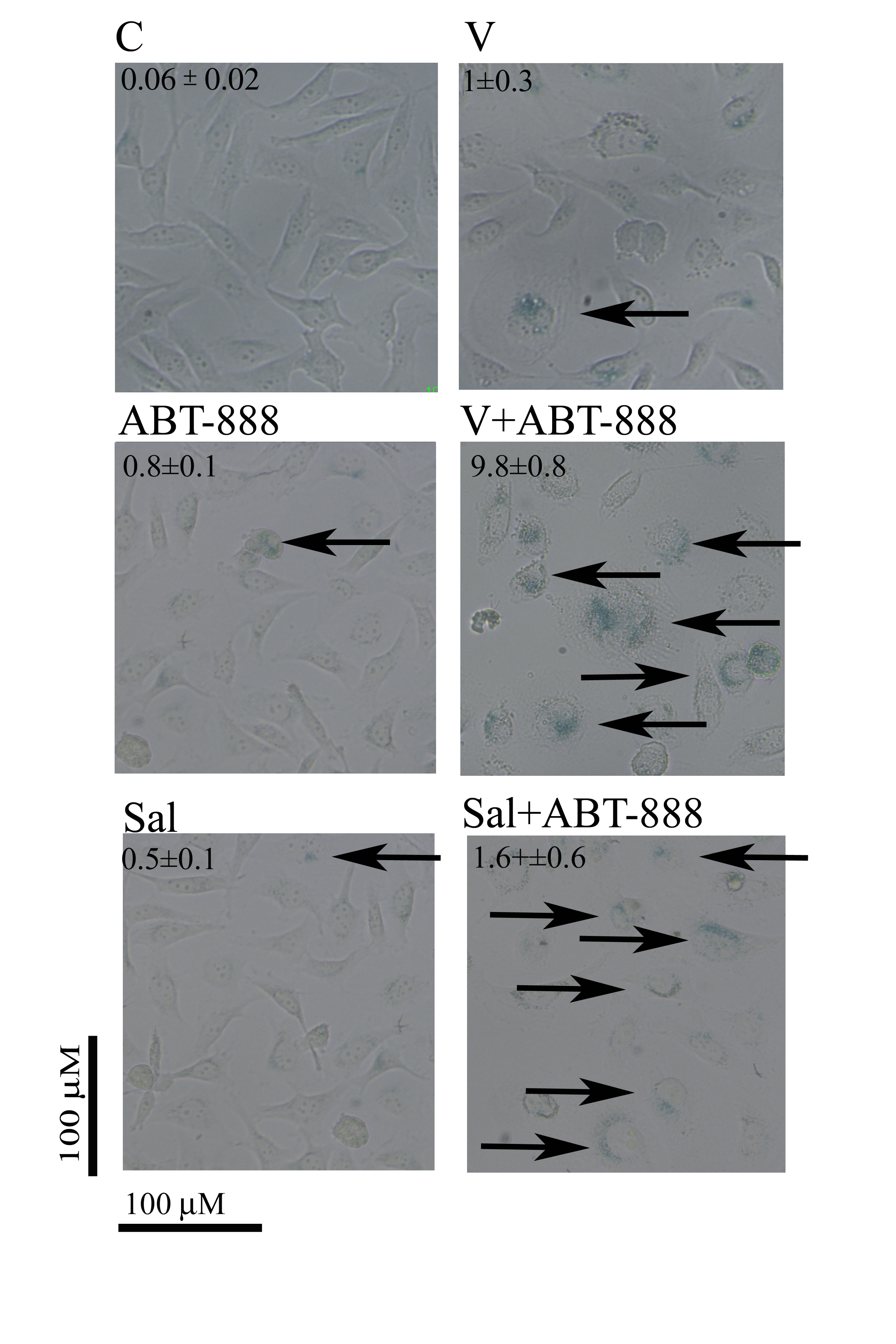

Supplement: S6 Fig — Cells were plated for colony survival assay and incubated with vorinostat (0.5 uM), ABT-888 (10 uM) vorinostat and ABT-888, salubrinal (4.5 uM) and salubrinal and ABT-888. Control received the vehicle. Cells were stained for senescence associated β-galactosidase activity (reflected in the blue stain). Numbers are average of senescent looking cells/colony ± SEM in triplicate plates and differences between the combined treatment and each one of the sole treatments or the control was statistically significant p<0.01. (TIF) [file pone.0155711.s006.tif]

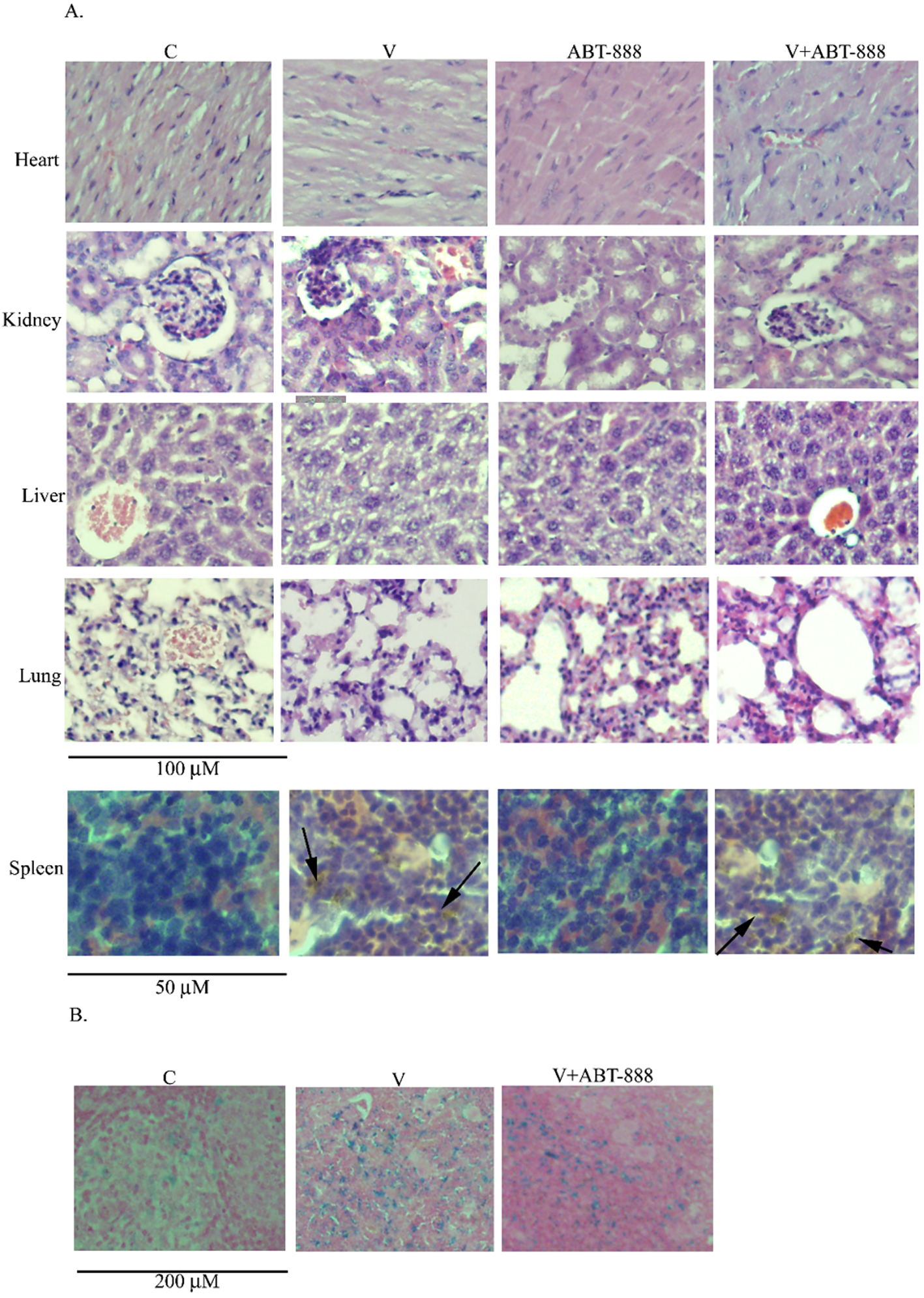

Supplement: S7 Fig — a. Sections were stained with H&E: The arrows point to brown stained areas in the spleen. b. Sections stained with Prussian blue: Increased blue staining is noted in spleen from vorinostat and vorinostat and ABT-888 treated mice. (TIF) [file pone.0155711.s007.tif]

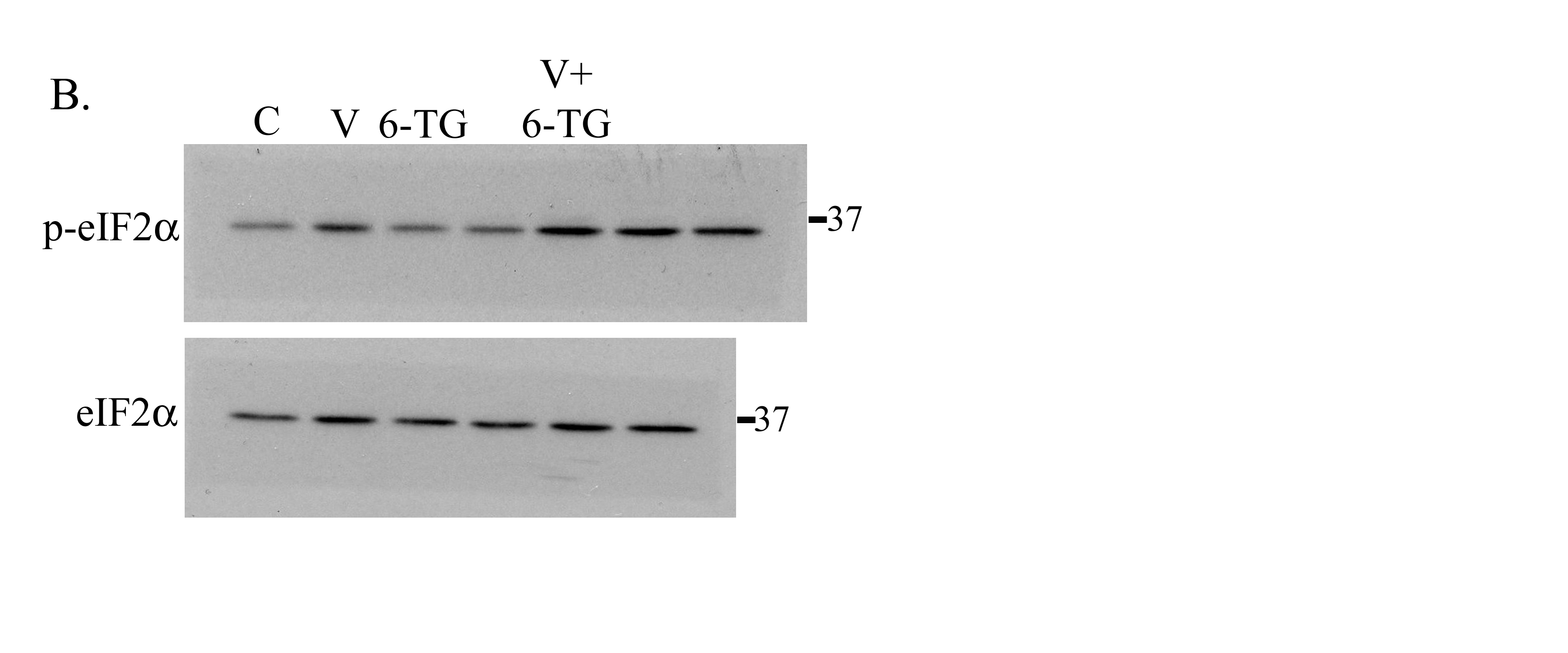

Supplement: S8 Fig — (TIF) [file pone.0155711.s008.tif]

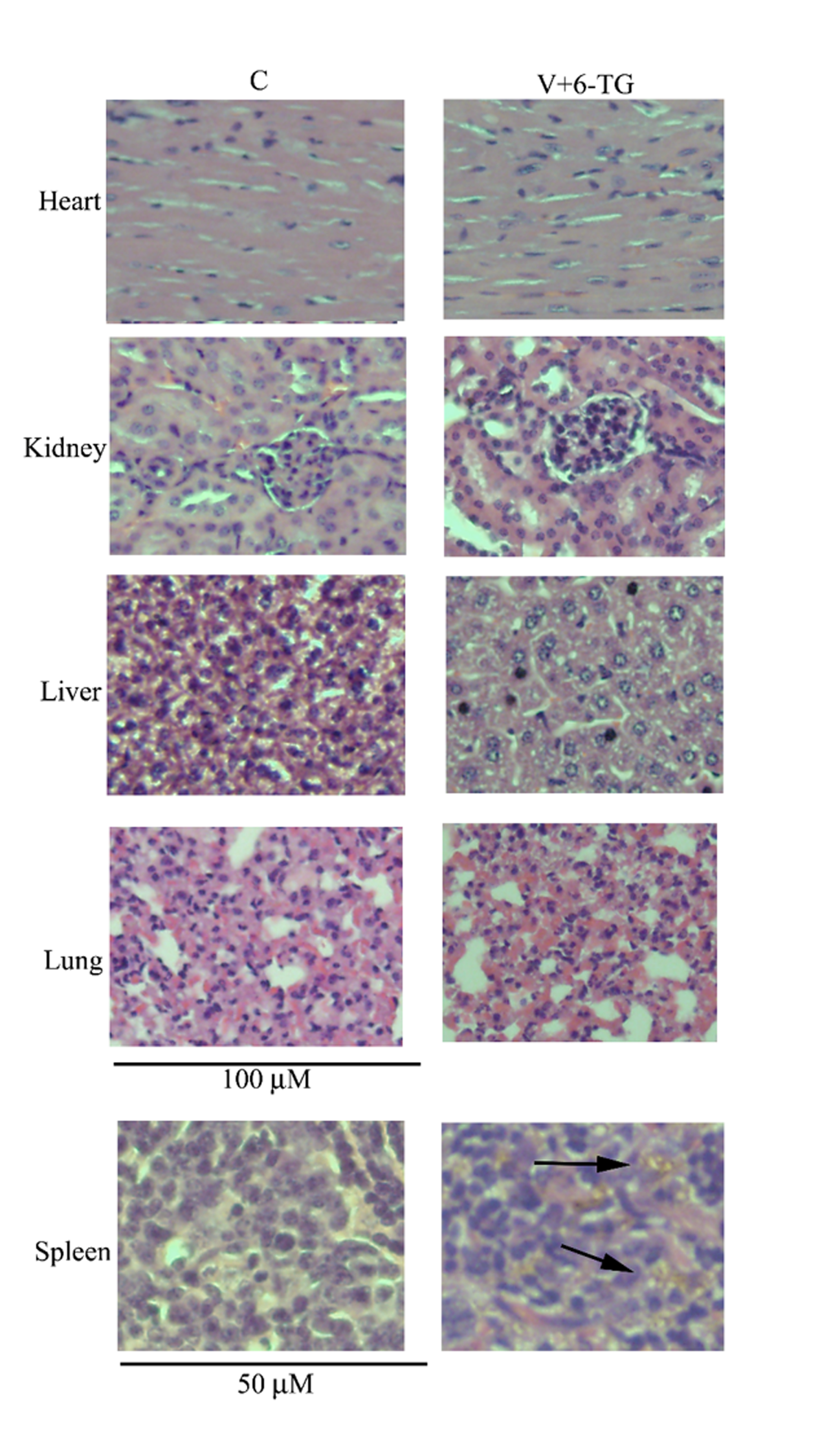

Supplement: S9 Fig — Sections were stained with H&E. The arrows point to brown stained areas in the spleen. (TIF) [file pone.0155711.s009.tif]
