# Supplementary material for: Overcoming Resistance of Cancer Cells to PARP-1 Inhibitors with Three Different Drug Combinations
Source: PLoS One. 2016 May 19;11(5):e0155711. doi: 10.1371/journal.pone.0155711 (PMC4873128; doi:10.1371/journal.pone.0155711)
Supplement: S1 Table — Cells plated for clonogenic survival assays were treated with vorinostat and ABT-888 at the specified ratios of vorinostat to ABT-888 as indicated in the table and in Fig 1. CI at effective doses of the drug combinations that leads to 50%, 75%, 90% and 95% clonogenic death were derived by employing the computer program CompuSyn. The low (< 0.9) CI values indicate synergistic interaction. (PDF) [file pone.0155711.s010.pdf]

**Cell Lines**	**V:ABT-888**	**ED50**	**ED75**	**ED90**	**ED95**
**MDA-MB-231**	1:14	0.53	0.45	0.39	0.36
**BT-549**	1:32	0.8	0.67	0.56	0.50
**MCF-7**	1:4	0.59	0.60	0.66	0.70
**U-87**	1:20	0.42	0.35	0.30	0.26
