# Supplementary material for: Overcoming Resistance of Cancer Cells to PARP-1 Inhibitors with Three Different Drug Combinations
Source: PLoS One. 2016 May 19;11(5):e0155711. doi: 10.1371/journal.pone.0155711 (PMC4873128; doi:10.1371/journal.pone.0155711)
Supplement: S2 Table — Cells plated for clonogenic survival assays were treated with salubrinal and ABT-888 at the specified ratio as described in Fig 3B. CI at effective doses of the drug combinations that leads to 50%, 75%, 90% and 95% clonogenic death were derived by employing the computer program CompuSyn. The low (< 0.9) CI values indicate synergistic interaction. (PDF) [file pone.0155711.s011.pdf]

**ABT-888: Salubrinal**	**ED50**	**ED75**	**ED90**	**ED95**
**1:1.5**	0.83	0.67	0.62	0.62
