# Supplementary material for: Overcoming Resistance of Cancer Cells to PARP-1 Inhibitors with Three Different Drug Combinations
Source: PLoS One. 2016 May 19;11(5):e0155711. doi: 10.1371/journal.pone.0155711 (PMC4873128; doi:10.1371/journal.pone.0155711)
Supplement: S3 Table — Mice were treated with 200 mg/kg vorisnotat, 40 mg/kg ABT-888 or their combinations. Controls received the vehicle. Blood was drawn by cardiac puncture from randomly sampled mice of each experimental group for determination of their hematocrit. * According to A.M.L. C-Control, V-vorinostat. (PDF) [file pone.0155711.s012.pdf]

	C	C	V	V	ABT-888	ABT-888	ABT-888 +V	ABT-888 +V	Normal range*
**WBC (10*3/µl)**	19.9	6.2	4.1	10.7	8.7	8.4	8.1	14.2	**3.2-12.7**
**HGB (g/dl)**	14.6	14.3	12.3	13.5	15	14.6	15.1	13.6	**11.8-14.9**
**Hematocrit (%)**	45.2	46.8	42	44.4	49.2	49.2	53.2	45.7	**36.7-46.8**
**MCV (fL)**	56.7	57.1	65.6	68.6	56	60.4	66.6	64.9	**42.2-59.2**
**Neutroph (%)**	70	24	48	64	49	57	59	70	**7-31**
**Lymph (%)**	30	76	52	35	51	41	41	30	**70-95**
**Platelets (10*3/µl)**	1447	933	924	1318	767	201	868	1535	**766-1659**
