# Supplementary material for: Overcoming Resistance of Cancer Cells to PARP-1 Inhibitors with Three Different Drug Combinations
Source: PLoS One. 2016 May 19;11(5):e0155711. doi: 10.1371/journal.pone.0155711 (PMC4873128; doi:10.1371/journal.pone.0155711)
Supplement: S4 Table — Mice were treated with 200 mg/kg vorisnotat, 40 mg/kg ABT-888 or their combinations. Controls received the vehicle. Blood was drawn by cardiac puncture from randomly sampled mice of each experimental group for determination of their hematocrit. * According to A.M.L. C-Control, V-vorinostat. (PDF) [file pone.0155711.s013.pdf]

	C	C	V	V	ABT-888	ABT-888	ABT-888 +V	ABT-888 +V	Normal range*
**Creatinine mg/dl**	0.15	0.13	0.21	0.09	0.12	0.15	0.21	0.13	**0.1-0.4**
**Calcium mg/dl**	10.9	10.3	11.6	11.2	10.2	11.1	10.6	11.4	**8.3-10.9**
**Phosphate mg/dl**	9.8	13.1	10.8	11.6	8.3	11.7	9.1	8.1	**5.2-13**
**Glucose mg/dl**	121	138	150	75	176	289	201	155	**46-237**
**Urea mg/dl**	46.1	35.9	35.1	51.3	42	39.9	53.4	46.8	**28-76**
**Cholesterol mg/dl**	141	115	136	106	152	154	147	95	**51-148**
**Protein g/dl**	5.53	5.13	6.06	5.79	5.55	5.76	6.18	6.05	**4.5-6.8**
**Albumin g/dl**	2.4	3	3.3	3.1	3.5	2.9	2.8	2.7	**2-2.4**
**Globulin g/dl**	3.1	2.1	2.8	2.7	2.1	2.9	3.4	3.3	**1.7-4.4**
**Total Bilirrubin mg/dl**	0.08	0.09	0.03	0.11	0.07	0.9	0.75	0.06	**0.1-0.3**
**Alkaline Phos IU/L**	39	114	51	41	71	69	60	39	**56-356**
**SGOT IU/L**	122	79	140	69	80	99	118	81	**38-361**
**SGPT IU/L**	36	31	36	17	31	33	35	18	**22-296**
**Sodium mmol/L**	161	156	160	160	158	158	163	160	**149-167**
**Potassium mmol/L**	9.1	9.6	8.6	9.7	7.3	10.3	7.8	7.4	**4.9-9.5**
**Cholesterol mmol/L**	107	109	110	108	108	106	109	106	**106-123**
