# Supplementary material for: Overcoming Resistance of Cancer Cells to PARP-1 Inhibitors with Three Different Drug Combinations
Source: PLoS One. 2016 May 19;11(5):e0155711. doi: 10.1371/journal.pone.0155711 (PMC4873128; doi:10.1371/journal.pone.0155711)
Supplement: S5 Table — Cells plated for clonogenic survival assays were treated with vorinostat and 6-TG at the specified ratio of as indicated in the table and in Fig 5. CI at effective doses of the drug combinations that leads to 50%, 75%, 90% and 95% clonogenic death were derived by employing the computer program CompuSyn. The low (< 0.9) CI values indicate synergistic interaction. (PDF) [file pone.0155711.s014.pdf]

Cell Lines	V:6-TG	ED50	ED75	ED90	ED95
**MDA-MB-231**	1:0.028	0.19	0.19	0.19	0.19
**BT-549**	1:10	0.72	0.70	0.67	0.66
**U-87**	1:4	0.19	0.24	0.29	0.35
