# Supplementary material for: Overcoming Resistance of Cancer Cells to PARP-1 Inhibitors with Three Different Drug Combinations
Source: PLoS One. 2016 May 19;11(5):e0155711. doi: 10.1371/journal.pone.0155711 (PMC4873128; doi:10.1371/journal.pone.0155711)
Supplement: S6 Table — Mice were treated with 150 mg/kg vorinostat, 0.75 mg/kg 6-TG or their combinations. Control received the vehicle. Blood was drawn by cardiac puncture from randomly sampled mice of [29] each experimental group for determination of their hematocrit. *According to A.M.L. C-Control, V-vorinostat. (PDF) [file pone.0155711.s015.pdf]

	C	V	6-TG	6-TG	V + 6-TG	V+ 6-TG	V + 6-TG	V+ 6-TG	normal range*
**WBC (10*3/µl)**	11.8	4.6	5.4	2.2	3.5	2.6	6.9	5	**3.2-12.7**
**HGB (g/dl)**	15	14.4	10.9	10.7	7.1	5	5.9	10	**11.8-14.9**
**Hematocrit (%)**	46	46.4	33.6	33.9	24.9	17.2	21.1	34.3	**36.7-46.8**
**MCV (fL)**	56.4	61.6	64.5	65.3	84.4	81.1	79.9	81.1	**42.2-59.2**
**Neutrophills (%)**	21	58	52	35	68	55	31	42	**7-31**
**Lymphocytes (%)**	76	40	45	62	28	43	69	58	**70-95**
**Platelets (10*3/µl)**	1259	1270	703	994	182	1109	935	1315	**766-1659**
