# Supplementary material for: Overcoming Resistance of Cancer Cells to PARP-1 Inhibitors with Three Different Drug Combinations
Source: PLoS One. 2016 May 19;11(5):e0155711. doi: 10.1371/journal.pone.0155711 (PMC4873128; doi:10.1371/journal.pone.0155711)
Supplement: S7 Table — Blood was drawn by cardiac puncture from randomly sampled mice of each experimental group for determination of blood chemistry. * According to A.M.L. SGOT- Aspartate aminotransferase, SPGT—Alanine transaminase. C-Control, V-vorinostat (150 mg/kg). 6-TG (0.75 mg/kg). (PDF) [file pone.0155711.s016.pdf]

	C	V	6-TG	V+6-TG	V+ 6-TG	V+ 6- TG	V+ 6-TG	Normal range*
**Creatinine mg/dl**	0.19	0.18	0.14	N.D	0.11	0.14	0.14	**0.1-0.4**
**Calcium mg/dl**	9.8	9.9	9.9	9.4	9.85	10.5	10.8	**8.3-10.9**
**Phosphate mg/dl**	8.3	9	12.7	10	12.1	6.9	9.7	**5.2-13**
**Glucose mg/dl**	114	145	135	124	125	225	277	**46-237**
**Urea mg/dl**	46	65.6	55.6	53.6	61.2	64.5	64.1	**28-76**
**Cholesterol mg/dl**	120	103	121	104	103	114	97	**51-148**
**Protein g/dl**	5.31	5.3	5.31	4.46	4.11	5.59	5.55	**4.5-6.8**
**Albumin g/dl**	3.7	3.5	3.6	2.7	2.6	3.9	3.8	**2-2.4**
**Globulin g/dl**	1.6	1.8	1.7	1.7	1.5	1.7	1.75	**1.7-4.4**
**Total Bilirrubin mg/dl**	0.1	0.07	0.07	0.56	0.05	0.08	0.1	**0.1-0.3**
**Alkaline Phosphatase IU/L**	99	94	81	54	35	84	83	**56-356**
**SGOT IU/L**	116	111	73	206	219	335	71	**38-361**
**SGPT IU/L**	26	34	22	36	37	34	14	**22-296**
**Sodium mmol/L**	155	154	151	150	156	151	154	**149-167**
**Potassium mmol/L**	6.2	5.8	7	6.8	6.9	9.9	9.1	**4.9-9.5**
**Cholesterol mmol/L**	109	106	110	100	113	115	108	**106-123**
